# Characteristics, treatment and survival of patients with chondrosarcoma in five European countries: a DARWIN EU^®^ cohort study

**DOI:** 10.2340/1651-226X.2026.45117

**Published:** 2026-03-10

**Authors:** Anton Barchuk, Cesar Barboza, Julieta Politi, Berta Raventós, Peter Prinsen, Jelle Evers, Vincent K.Y. Ho, Michiel A.J. van de Sande, Eric Fey, Kimmo Porkka, Anna Hammais, Tiina Wahlfors, Tuomo Nieminen, Toni Lehtonen, Antonella Delmestri, Guillaume Verdy, Romain Griffier, Airam de Burgos-González, Ana Llorente-Garcia, Cristina Justo-Astorgano, Miguel-Angel Macia-Martinez, Anja Schiel, Olli Tenhunen, Alexandra Pacurariu, Ross Brennan, Ross Williams, Katia Verhamme, Talita Duarte Salles

**Affiliations:** a Department of Medical Informatics, Erasmus Medical Center, Rotterdam, The Netherlands; b University of Helsinki, Helsinki, Finland; c Netherlands Comprehensive Cancer Organisation (IKNL), Utrecht, The Netherlands; d Orthopedic Surgery, Leiden University Medical Center, Leiden, The Netherlands; e iCAN Digital Precision Cancer Medicine Flagship, University of Helsinki and Helsinki University Central Hospital Cancer Center, Helsinki, Finland; f Finnish Institute for Health and Welfare (THL), Helsinki, Finland; g Centre for Statistics in Medicine, Nuffield Department of Orthopaedics, Rheumatology, and Musculoskeletal Sciences (NDORMS), University of Oxford, Oxford, United Kingdom; h Public Health Department, Medical Information Service, University Hospital of Bordeaux, Bordeaux, France; i Agencia Española de Medicamentos y Productos Sanitarios, Madrid, Spain; j Norwegian Medical Products Agency (NOMA), Oslo, Norway; k Medical Research Center Oulu, Oulu University Hospital, University of Oulu, Oulu, Finland; l Finnish Medicines Agency, Helsinki, Finland; m Real World Evidence Workstream, European Medicines Agency, Amsterdam, The Netherlands; n Fundació Institut Universitari per a la recerca a l’Atenció Primària de Salut Jordi Gol i Gurina (IDIAPJGol), Barcelona, Spain

**Keywords:** Chondrosarcoma, survival, federated analysis, rare cancers, Darwin EU

## Abstract

**Background and purpose:**

Chondrosarcoma is a rare bone malignancy with a poor response to systemic therapy in advanced stages. European-level epidemiological data remain scarce. This study aimed to characterise patient demographics, treatments and survival using real-world data to inform regulatory decisions about the feasibility and design of new trials for the systemic treatment of chondrosarcoma.

**Patient/material and methods:**

This cohort study, part of the DARWIN EU^®^ initiative, analysed data from six healthcare databases in Finland, France, the Netherlands, Spain and the UK. Patients diagnosed with chondrosarcoma between 2010 and 2022 were identified. Standardised analyses were performed within a federated network using the Observational Medical Outcomes Partnership (OMOP) Common Data Model.

**Results:**

A total of 2,498 chondrosarcoma patient records were identified, covering at least 2,356 unique patients. Median age at diagnosis was 52–55 years, with a balanced sex distribution. Surgical treatment was the most common intervention, recorded in 15.2% to 88.9% of patients, depending on the database. Fewer than 5% received systemic anticancer therapy, and radiotherapy was reported in fewer than 7%. The 10-year overall survival (OS) ranged from 58% (95% confidence interval [CI]: 43–78) to 80% (95% CI: 78–82), with restricted mean survival between 7.4 and 8.7 years. In the Netherlands, patients with late-stage, metastatic or high-grade disease showed significantly poorer outcomes.

**Interpretation:**

This study demonstrates the feasibility of using real-world data across Europe to describe chondrosarcoma patients. Most had early-stage, low-grade disease amenable to surgery, with limited use of systemic therapies. Survival was generally favourable, except in advanced disease. Clinical trials remain difficult due to the rarity of advanced chondrosarcoma and the lack of standards.

## Introduction

Chondrosarcoma is a relatively rare cancer, but along with osteosarcoma, it represents the majority of primary bone malignancies [1]. Conventional chondrosarcoma accounts for approximately 75% of all chondrosarcoma cases, while dedifferentiated and mesenchymal chondrosarcoma represent rare and highly aggressive variants [2]. Several national epidemiological studies have specifically examined the population-based incidence of chondrosarcoma, ranging from 0.27 to 5.4 cases per million per year [3]. The majority of patients diagnosed with chondrosarcoma have been reported to be diagnosed with Grade 1 tumours and early-stage disease. In the Netherlands, an increase in the number of radiological examinations has been partially attributed to the increased incidence of Grade 1 tumours [4]. Several changes to classification might have affected the epidemiology of chondrosarcoma. The term ‘atypical cartilaginous tumours’ was introduced in 2013 [5] and initially aligned with chondrosarcoma Grade 1. In the 2020 classification, it was separated from Grade 1 chondrosarcoma and is now considered a relatively benign condition [6].

Surgery is the primary treatment for chondrosarcoma with curative intent. Still, distant metastases can be detected more than 10 years after the initial treatment [7]. Most late-stage chondrosarcomas are resistant to standard anticancer therapies, and treatment options are limited in patients with metastatic or unresectable tumours [8]. A study based on data from the National Cancer Database (NCD) in the United States showed only a slight improvement in the overall survival (OS) of patients with chondrosarcoma in recent periods (2004–2015) [9].

Chondrosarcoma’s relative rarity and limited number of studies make it challenging to have a clear picture across Europe of the characteristics of these patients at the time of diagnosis, the therapy they receive and their OS [10]. Thus, it is challenging to plan clinical trials that could focus on drug therapy for chondrosarcoma. This study aimed to inform these aspects, which are also important from a regulatory point of view, to provide context and help understand how new medicines may add value for chondrosarcoma patients.

## Patients/material and methods

This cohort study was conducted within the DARWIN EU^®^ [11, 12] project, which is run by the coordinating centre at Erasmus University Medical Center in Rotterdam and established by the European Medicines Agency (EMA) and the European Medicines Regulatory Network. In this study, we used routinely collected health data from six nationwide or region-wide databases in five European countries.

The study population comprised all individuals with a first diagnosis of chondrosarcoma identified in each database between January 1, 2010, and December 31, 2022, to allow at least 1 year of potential follow-up. Participants with a diagnosis of cancer (any, excluding non-melanoma skin cancer) before the diagnosis of chondrosarcoma were excluded.

Heterogeneous data sources were selected for this study to examine diverse populations and healthcare settings. The following data sources were used: Base de Datos para la Investigación Farmacoepidemiológica en el Ámbito Público (BIFAP), Spain; Clinical Data Warehouse of Bordeaux University Hospital (CDWBordeaux), France; Clinical Practice Research Datalink GOLD (CPRD GOLD), UK; Finnish Care Register for Health Care (FinOMOP-HILMO), Finland; Hospital District of Helsinki and Uusimaa (FinOMOP-HUS), Finland; Netherlands Cancer Registry (NCR), the Netherlands. Our study aimed to describe chondrosarcoma patients in various data sources and healthcare settings, including primary healthcare (CPRD GOLD and BIFAP), secondary and tertiary care (CDWBordeaux and FinOMOP-HUS), a national hospital registry (FinOMOP-HILMO) and a national cancer registry (NCR). BIFAP is a database based on primary and secondary healthcare records from nine of the 17 regions in Spain, covering ~22 million individuals. CDWBordeaux is the Bordeaux University Hospital database, which includes records of ~2.2 million individuals. FinOMOP-HILMO is a nationwide hospital discharge records registry. At the time of this study, the dataset included records for ~7.3 million individuals. FinOMOP-HUS is a hospital registry dataset from the Uusimaa region of Finland, with records for 3.5 million individuals. NCR is a nationwide cancer registry of the Netherlands, with records of ~2.5 million individuals. Details on data sources are provided in the Supplementary Material (Table S1). Data completeness for cancer patients was high in nationwide registries – FinOMOP-HILMO [13] and NCR [14]. However, it was reported to be lower for some cancer types in the primary healthcare data source CPRD GOLD [15].

All databases were mapped to the Observational Medical Outcomes Partnership (OMOP) Common Data Model (CDM). This enabled the use of standardised analytics and tools across the DARWIN EU^®^ network, as the structure of the data and the terminology system were harmonised [16]. The OMOP CDM is developed and maintained by the Observational Health Data Sciences and Informatics (OHDSI) initiative [17]. Each data partner executed the analytical code (created in *R*) against their patient-level data and returned a results set, which only contained aggregated data. No sample size calculation was performed as this was a descriptive study, and we were interested in the characteristics of all newly diagnosed chondrosarcoma patients within the study period.

Descriptive analytics (mean, median, interquartile range [IQR] and proportions) were used to describe the age and sex of patients at diagnosis, as well as the treatments, including medications, surgical procedures and radiotherapy. We further stratified results from NCR by American Joint Committee on Cancer/Union for International Cancer Control (AJCC/UICC) stage and Tumor, Node, Metastasis (TNM) categories, histological subtypes, grades and tumour site.

The study population (chondrosarcoma patients), variables used for stratifying results, as well as all medications and procedures, were defined using standard concepts from the OMOP standardised vocabularies during the phenotyping process [11, 17, 18]. To define chondrosarcoma, we used relevant Systematised Nomenclature of Medicine – Clinical Terms (SNOMED) and the International Classification of Diseases for Oncology, 3^rd^ Edition (ICD-O-3) terms (Table S2), RxNorm terminology-based concepts were used to define specific drug (Table S3) and SNOMED-based concepts were used to determine surgical and radiological procedures (Table S4). Therapy options were not collected and thus not assessed in the CPRD GOLD and BIFAP databases. Medication use was assessed in three time windows: from diagnosis date to 90 days after diagnosis, 91–365 days after diagnosis and 365 days after diagnosis. Surgical and radiotherapy procedures were assessed from the date of diagnosis to 180 days after diagnosis. For the NCR, treatment and procedures 365 days after diagnosis were not available, and patients younger 18 were also not included in NCR.

The Kaplan-Meier (KM) method was used to estimate OS, utilising data on the time at risk of death from any cause. Results were reported using medians, restricted mean survival time (RMST) for the 10-year follow-up with standard error (SE) and survival probabilities at 1, 3, 5 and 10 years with 95% confidence intervals (CIs). A formal comparison or meta-analysis was not planned due to data heterogeneity; therefore, results were presented separately for each data source.

We used the R packages *PatientProfiles* [19] for the patient-level characterisation of demographics and description of treatments, and *CohortSurvival* [20] to estimate OS.

## Results

Chondrosarcoma patients were identified in all six databases: 379 in BIFAP, 92 in CDWBordeaux, 54 in CPRD GOLD, 382 in FinOMOP–HILMO, 142 in FinOMOP–HUS and 1,449 in NCR. Attrition of individuals included in the study and its contribution to the study objectives are presented in Table S5. Demographic characteristics of patients are presented in Table 1. The median age of patients with chondrosarcoma ranged between 52 and 55 years in FinOMOP-HILMO and FinOMOP-HUS, respectively. The proportion of patients aged 60 or older ranged from 44% in FinOMOP-HILMO to 35% in NCR. Overall, the proportion of men and women was similar across all databases.

**Table 1:** Demographic characteristics of patients with chondrosarcoma from 2010 to 2022.

Demographic characteristics		BIFAP	CDW Bordeaux	CPRD GOLD	FinOMOP - HILMO	FinOMOP - HUS	NCR
*N*		379	92	54	382	142	1,449
Sex, *N* (%)	Female	204 (54%)	41 (45%)	28 (52%)	179 (47%)	70 (49%)	733 (51%)
	Male	175 (46%)	51 (55%)	26 (48%)	203 (53%)	72 (51%)	716 (49%)
Age, Median [Q25-Q75]		53 [42–68]	54 [36–66]	53 [42–66]	55 [40–68]	52 [37–67]	53 [43–64]
Age, Mean (SD)		54.3 (16.9)	51.7 (19.4)	51.5 (17.6)	54.2 (18.6)	51.5 (19.2)	52.9 (15.5)
Age, Range		4 to 92	15 to 92	11 to 92	8 to 93	8 to 92	18 to 93
Age group (years), *N* (%)	0 to 19	8 (2.1%)	NA (NA%)	NA (NA%)	13 (3.4%)	6 (4.2%)	12 (0.8%)
	20 to 39	69 (18.2%)	25 (27.2%)	11 (20.4%)	81 (21.2%)	36 (25.4%)	281 (19.4%)
	40 to 59	159 (42.0%)	30 (32.6%)	22 (40.7%)	120 (31.4%)	48 (33.8%)	656 (45.3%)
	60 to 79	119 (31.4%)	27 (29.3%)	16 (29.6%)	142 (37.2%)	42 (29.6%)	439 (30.3%)
	80+	24 (6.3%)	19 (10.9%)	5 (9.3%)	26 (6.8%)	10 (7.0%)	61 (4.2%)

SD: standard deviation; FinOMOP-HILMO: Finnish Care Register for Health Care; FinOMOP-HUS: Hospital District of Helsinki and Uusimaa Database; NCR: the Netherlands Cancer Registry.

No drug treatment was identified among patients with chondrosarcoma at CDWBordeaux. Less than 5% of patients had a record of a prescription for any specific therapy across the other three databases (NCR, FinOMOP-HUS and FinOMOP-HILMO), with most records for conventional chemotherapy. Table 2 describes medication drug groups. Only the following drugs were identified: Carboplatin (NCR), Cisplatin (NCR and FinOMOP-HUS), Cyclophosphamide (NCR), Dasatinib (NCR), Docetaxel (NCR and FinOMOP-HUS), Durvalumab (NCR), Etoposide (NCR), Gemcitabine (NCR and FinOMOP-HUS), Irinotecan (NCR), Methotrexate (NCR, FinOMOP-HUS and FinOMOP-HILMO), Nivolumab (NCR), Pazopanib (NCR and FinOMOP-HUS), Regorafenib (FinOMOP-HILMO), Temozolomide (NCR and FinOMOP-HILMO) and Vincristine (NCR). For most identified drugs, the number of records was less than 5.

**Table 2:** Treatment of patients with chondrosarcoma in different time windows after chondrosarcoma diagnosis.

Period	Type of therapy	FinOMOP - HILMO	FinOMOP - HUS	NCR
		382	142	1,449
0 to 90 days	Conventional chemotherapy	< 5	< 5	6 (0.4%)
	Tyrosine kinase inhibitors	–	–	< 5
91 to 365 days	Conventional chemotherapy	5 (1.3%)	6 (4.2%)	12 (0.8%)
	Tyrosine kinase inhibitors	–	–	< 5
More than 365 days^1^	Conventional chemotherapy	6 (1.6%)	5 (3.5%)	–
	Programmed cell death protein 1 inhibitors	–	–	–
	Tyrosine kinase inhibitors	< 5	< 5	–

FinOMOP-HILMO: Finnish Care Register for Health Care; FinOMOP-HUS: Hospital District of Helsinki and Uusimaa Database; NCR: the Netherlands Cancer Registry.

1 Not reported for NCR as likely to be incomplete.

Surgical procedures were identified only in the records of 15.2% of patients in CDWBordeaux, compared with 65.2% in FinOMOP-HILMO, 53.5% in FinOMOP-HUS and 88.9% in NCR. FinOMOP – HILMO, FinOMOP – HUS and NCR radiotherapy records were also identified, with a maximum of 6.4% of patients at NCR (Table S6).

Additional information on tumour grade, stage, TNM categories and the anatomical site was available only in NCR. Most chondrosarcoma cases were diagnosed as Stage I (54.9%) and, more specifically, Stage IA (47.0%), whilst Stage IV was present only in 3.7% of patients. Nodal metastases (N+) within 180 days after diagnosis were recorded in 1.4% of patients and distant (M1) in 4.1% of patients (Table 3). Most patients had tumours of longer bones of the lower (41.3%) and upper limbs (18.2%), followed by rib, sternum and clavicle tumours (11.3%) (Table S7). Patients with non-conventional chondrosarcoma (*n* = 215) had dedifferentiated (*n* = 94, 44.8%), myxoid (*n* = 71, 33.0%) or periosteal tumours (*n* = 28, 13.0%), and about 5% (*n* = 11) had clear cell or mesenchymal chondrosarcoma.

**Table 3:** Overall 1, 3, 5 and 10-year survival of patients with chondrosarcoma by data source.

Estimates	BIFAP	CDW Bordeaux	CPRD GOLD	FinOMOP - HILMO	FinOMOP - HUS	NCR
Records in the analysis	379	92	54	382	137	1,448
Number events	63	21	9	100	34	243
Restricted mean survival (years) (SE)	8.5 (0.5)	7.4 (0.5)	8.1 (0.6)	8.0 (0.2)	7.5 (0.4)	8.7 (0.1)
1 year survival, % (95% CI)	94 (91, 96)	92 (87, 98)	90 (82, 99)	91 (88, 94)	89 (84, 95)	94 (93, 95)
3 year survival, % (95% CI)	88 (84, 91)	78 (68, 89)	85 (76, 96)	83 (79, 87)	80 (73, 88)	90 (88, 91)
5 year survival, % (95% CI)	84 (80, 88)	68 (57, 82)	82 (71, 95)	79 (75, 83)	76 (69, 85)	86 (84, 88)
10 year survival, % (95% CI)	79 (74, 84)	58 (43, 78)	73 (55, 95)	68 (63, 74)	61 (50, 75)	80 (78, 82)

SE: standard error; CI: confidence interval; FinOMOP-HILMO: Finnish Care Register for Health Care; FinOMOP-HUS: Hospital District of Helsinki and Uusimaa Database; NCR: the Netherlands Cancer Registry.

NR – Median survival was not reached in all databases (more than 50% of patients were still alive by the end of the follow-up).

**Figure 1:**
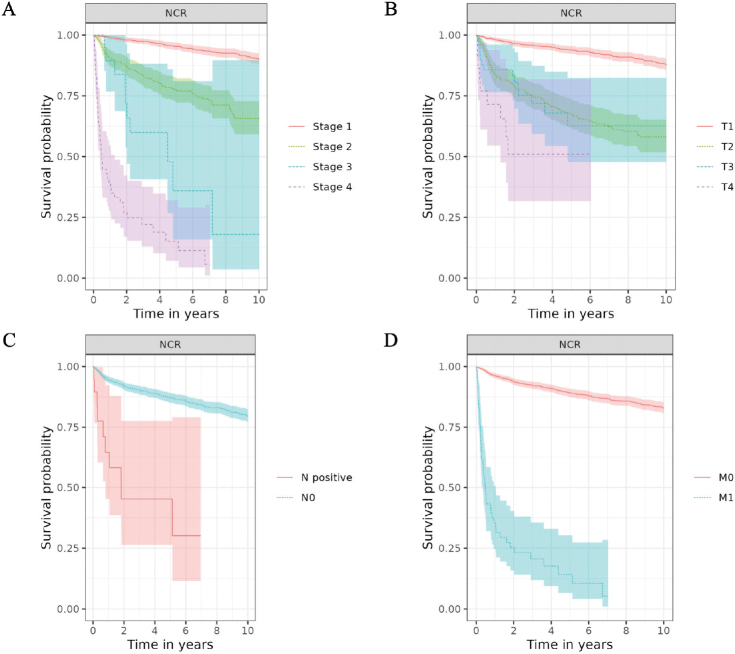
Overall survival of patients with chondrosarcoma by Stage (A) and T (B), N (C) and M (D) categories of AJCC/UICC TNM staging classification.

The median follow-up was 3 years for CPRD GOLD, 4 years for CDWBordeaux and FinOMOP-HUS, 6 years for BIFAP, 8 years for FinOMOP-HILMO and 9 years for NCR. Median survival was not reached in any of the databases (meaning that more than 50% of patients were still alive by the end of the follow-up). The 10-year survival probability ranged between 58% (95% CI: 43, 78) in CDWBordeaux and 80% (95% CI: 78, 82) in NCR (Table 3 and Figure 1).

**Figure 2:**
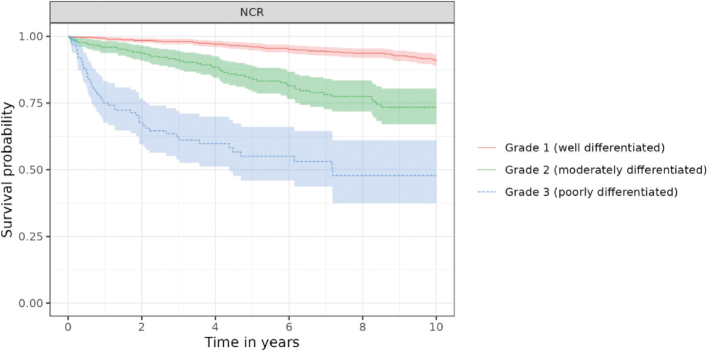
Overall survival of patients with chondrosarcoma by Grade in NCR.

In NCR, survival estimates were consistent with the stage categories: Stage I patients had a 95% 5-year survival probability, and Stage IV patients had 15% (Table S7). Similarly, survival estimates aligned with TNM categories, with the lowest survival estimates for patients with T4, N+ and M1 tumours (Table S8). Survival estimates were consistently lower for patients with axial skeleton tumours than for those with extremity tumours, with the highest estimates for short and long bones of the upper and lower limbs, and for face and skull tumours. The 10-year OS probability was 91% (89, 93) for Grade 1 and only 48% (37, 61) for Grade 3 (Figure 2, Table S9).

Survival estimates for non-conventional chondrosarcoma were substantially lower for dedifferentiated and mesenchymal tumours than for other histological subtypes (Table S9).

## Discussion

Chondrosarcoma is a rare condition, and studies describing the characteristics of patients in large, representative databases are uncommon. Our study included over 2,000 cases from six databases in five European countries between 2010 and 2022. Medication use was sporadic across all databases, and OS was relatively high, except for specific subtypes and late-stage disease identified in the Dutch cancer registry data.

Only a few studies have been conducted previously to describe patients with chondrosarcoma. A Dutch study, which utilised the NCR data between 1989 and 2013, identified 2,186 chondrosarcoma patients and described patient and tumour characteristics, as well as their relationship to OS [4]. Our findings for patients identified between 2010 and 2022 were in line with the results of the previous study. Even though we excluded atypical cartilaginous tumours, Grade 1 and Stage I chondrosarcoma remain the most common diagnosis in the Netherlands and probably across Europe, given comparable survival across all databases. Several other studies have described the patient and tumour characteristics, demographics and survival of chondrosarcoma patients in the NCD in the United States, with the latest study covering 5,329 chondrosarcoma patients from 2004 to 2015 [9]. In this study, metastatic disease was present at diagnosis for 6.2% of patients, and it was 4.1% in the NCR in our study.

The demographics of chondrosarcoma patients across all databases were similar, indicating that hospital-based and primary care data sources provide reliable information on newly diagnosed cases of chondrosarcoma. The median age ranged from 53 to 55 years, with an equal proportion of men and women, consistent with other studies [7, 9].

Whilst information on surgical treatment was available in all databases that reported treatment for chondrosarcoma, radiotherapy and, more specifically, drug treatments were rare. Our study identified around 5% of patients with records of any drug treatment, aligning with previous studies. The NCD study identified drug treatment in fewer than 7% of chondrosarcoma patients [9]. The majority of patients with conventional chondrosarcoma had early-stage and low-grade disease, which is also in line with previous studies and may partially explain the lack of information on drug therapy. In the NCR, the proportion of distant metastases was below 5%, but metastases were more common in patients with non-conventional chondrosarcoma. The wide variation in surgical management (15%–89%) we observed may reflect differences in populations, but it is also likely to reflect differences in data capture and granularity. The lack of standardisation in the description of surgical procedures calls for additional effort to harmonise the capture of surgical information.

Regarding the use of cancer medication in chondrosarcoma patients, the most common agents represented conventional chemotherapy. Still, some records of immunotherapies and targeted agents were also identified, including durvalumab, nivolumab, dasatinib, pazopanib and regorafenib, which were potentially administered in the context of clinical trials. The use of most of these drugs has been previously described in published studies. Pazopanib and dasatinib were previously reported to be used in Phases 1 and 2 trials in patients [21–23] with chondrosarcoma. More recently, the use of regorafenib in patients with metastatic or locally advanced chondrosarcoma was tested in a Phase 2 randomised trial [24]. Finally, immunotherapy drugs have also been reported to be used in patients with chondrosarcoma [23, 25, 26]. The key problem for most reports of new drug agents in chondrosarcoma is that they are limited to small case series or trials with limited sample sizes [23]. Those drug therapies remain limited to off-label use in late-stage, high-grade and metastatic disease cases. Whilst this is a problem for most rare diseases, the analysis across multiple data sources we performed can help evaluate which specific populations and healthcare settings are suitable for planning future clinical trials.

OS was relatively high in all the databases and aligned with previous studies [4, 7, 9, 27, 28]. In the EUROCARE-6 study, chondrosarcoma survival was highest among all bone sarcomas in all age groups (5-year relative survival – 73%) [27], including adolescents and young adults in the EUROCARE-6 study (5-year relative survival – 87%) [28]. Our analysis revealed similar results. Subgroup analysis by stage, grade and anatomical site yielded results comparable to those of previous studies: survival was worse in patients with late-stage disease, in the presence of metastases and in those with high-grade disease [29, 30]. However, some variability in terms of survival existed between databases. Survival estimates were slightly lower in CDWBordeaux and FinOMOP-HUS, but higher in NCR and BIFAP. Differences in patients’ ages may partially explain the variations in survival estimates, with older age being one of the factors associated with poorer survival outcomes.

Additionally, general hospitals (in CDWBordeaux and FinOMOP-HUS) are more likely to admit more advanced cases than other healthcare settings, where patients with chondrosarcoma can be referred to. However, chondrosarcoma care is usually concentrated in highly specialised centres, given the rarity and complexity of the condition. Differences in OS between databases should be further explored.

Treatment information was available in three databases capturing hospital-based care but was absent from the primary healthcare database, indicating that the majority of chondrosarcoma patients receive systemic treatment in inpatient settings. We also did not find any records of systemic therapy use in CDWBordeaux, which could suggest incomplete recording; however, it may also indicate that the hospital is not providing care to patients with late-stage chondrosarcoma, a more plausible explanation. In the Nouvelle-Aquitaine region, the Institut Bergonié is the Comprehensive Cancer Centre responsible for treating patients with sarcoma.

This study is one of the first examples of using DARWIN EU^®^ to deliver real-world evidence on cancer from across Europe. Although data sources had some limitations, they were still representative of different healthcare settings and included a higher number of chondrosarcoma cases than the national reports. As the network of DARWIN EU^®^ data partners expands, and the amount of information mapped to OMOP CDM increases in each source, real-world evidence on cancer is becoming increasingly representative and granular. Federated analysis across multiple data sources can help identify populations and healthcare settings where clinical trials are feasible.

The study had several limitations. First, it was informed by routinely collected healthcare data, so data quality issues must be taken into consideration. In particular, the identification of chondrosarcoma patients varied across databases. Chondrosarcoma patient counts may be lower than expected, particularly for databases that lack patient-level linkage to secondary care data. Nevertheless, at least one database (NCR) provided high-quality cancer diagnosis data with a level of granularity comparable to that used in previously published studies on chondrosarcoma. At the same time, comparing results across multiple databases offers important insights for future oncology research in real-world settings. Although population-based cancer registries remain a high-quality source of cancer-specific information, they typically lack patient-level characteristics, such as comorbidities and medication use, which are available in other data sources. For example, details on treatment lines, treatment responses and tumour molecular characteristics can be found in established institutional cohorts and dedicated disease registries. Improving data quality and linkage outside cancer registries would substantially broaden the scope of real-world oncology research.

Second, Finnish databases were analysed independently, but an overlap between FinOMOP-HUS and FinOMOP-HILMO is likely. FinOMOP-HILMO is a national hospital discharge registry with high completeness for cancer patients [13]. FinOMOP-HUS, representing a university hospital with a comprehensive cancer centre, also contributes to FinOMOP-HILMO. Whilst patients identified in FinOMOP-HUS may partially overlap with those in FinOMOP-HILMO, we deliberately analysed both data sources because differences in data granularity and patient mix were expected between them.

Third, excluding patients with prior malignancies in our study was motivated by the aim of replicating the exclusion criteria used in clinical trials. It is preferable to avoid these exclusion criteria in a real-world data study to obtain a more comprehensive picture of real-world characteristics and outcomes in patients with chondrosarcoma. Additional attention may also be given to radiation-induced tumours that follow treatment for prior malignancies [31].

Fourth, the phenotyping process for describing surgical procedures and radiotherapy in chondrosarcoma was suboptimal, unlike systematic therapy that can be described at the ingredient level. The selection of concepts describing surgical procedures was based on all available procedures across databases and should be refined for future studies to specific procedures.

We also did not include patients registered in 2023 or later, so several agents introduced in recent years could not be identified [32, 33].

Importantly, this work identifies key gaps in data collection and reporting that are crucial for future studies of chondrosarcoma and, more broadly, cancer, including incomplete staging, insufficient treatment information and missing biomarker data. These gaps should be addressed in future DARWIN EU^®^ studies and other federated oncology research efforts.

A clear understanding of the scope and content of data captured from different sources is essential to strengthen real-world evidence generation, particularly for chondrosarcoma, where clinical trial designs and availability remain challenges.

## Conclusion

Different real-world data sources can be successfully utilised to study the outcomes and characteristics of rare cancer patients in the DARWIN EU^®^. Our study described the characteristics of patients with chondrosarcoma and their survival across several European countries, contributing to the overall evidence on this rare disease in the European population. Overall, treatment with medicines remains extremely rare in chondrosarcoma, explained by the fact that most patients have early-stage and low-grade disease that is amenable to curative surgical treatment. OS of chondrosarcoma patients remains relatively high, except for non-conventional chondrosarcoma and patients with high-grade and metastatic disease. Clinical trials of new drugs for chondrosarcoma remain a challenge, especially in the absence of standard treatment and a small overall number of patients with advanced disease.

## Supplementary Material



## Data Availability

Individual-level data are not available for this study. Detailed results of federated analysis are also available in a web application at https://data.darwin-eu.org/EUPAS1000000162/.

## References

[1] Valery PC, Laversanne M, Bray F. Bone cancer incidence by morphological subtype: a global assessment. Cancer Causes Control. 2015;26(8):1127–39. 10.1007/s10552-015-0607-3 26054913

[2] Gelderblom H, Hogendoorn PCW, Dijkstra SD, Van Rijswijk CS, Krol AD, Taminiau AHM, et al. The clinical approach towards chondrosarcoma. Oncologist. 2008;13(3):320–9. 10.1634/theoncologist.2007-0237 18378543

[3] Thorkildsen J, Myklebust TÅ. The national incidence of chondrosarcoma of bone; a review. Acta Oncol. 2023;62(2):110–7. 10.1080/0284186X.2023.2177975 36856035

[4] Van Praag (Veroniek) VM, Rueten-Budde AJ, Ho V, Dijkstra PDS, Fiocco M, Van De Sande MAJ, et al. Incidence, outcomes and prognostic factors during 25 years of treatment of chondrosarcomas. Surg Oncol. 2018;27(3):402–8. 10.1016/j.suronc.2018.05.009 30217294

[5] Suster D, Hung YP, Nielsen GP. Differential diagnosis of cartilaginous lesions of bone. Archiv Pathol Lab Med. 2020;144(1):71–82. 10.5858/arpa.2019-0441-RA 31877083

[6] Choi JH, Ro JY. The 2020 WHO classification of tumors of bone: an updated review. Adv Anat Pathol. 2021;28(3):119–38. 10.1097/PAP.0000000000000293 33480599

[7] Thorkildsen J, Taksdal I, Bjerkehagen B, Haugland HK, Børge Johannesen T, Viset T, et al. Chondrosarcoma in Norway 1990–2013; an epidemiological and prognostic observational study of a complete national cohort. Acta Oncol. 2019;58(3):273–82. 10.1080/0284186X.2018.1554260 30632866

[8] Polychronidou G, Karavasilis V, Pollack SM, Huang PH, Lee A, Jones RL. Novel therapeutic approaches in chondrosarcoma. Future Oncol. 2017;13(7):637–48. 10.2217/fon-2016-0226 28133974

[9] Ottesen TD, Shultz BN, Munger AM, Amick M, Toombs CS, Friedaender GE, et al. Chondrosarcoma patient characteristics, management, and outcomes based on over 5,000 cases from the National Cancer Database (NCDB). PLoS One. 2022;17(7):e0268215. 10.1371/journal.pone.0268215 35901087 PMC9333210

[10] Nota SPFT, Braun Y, Schwab JH, Van Dijk CN, Bramer JAM. The identification of prognostic factors and survival statistics of conventional central chondrosarcoma. Sarcoma. 2015;2015:1–11. 10.1155/2015/623746 PMC4655064 26633939

[11] Dernie F, Corby G, Robinson A, Bezer J, Mercade‐Besora N, Griffier R, et al. Standardised and reproducible phenotyping using distributed analytics and tools in the Data Analysis and Real World Interrogation Network (DARWIN EU). Pharmacoepidemiol Drug. 2024;33(11):e70042. 10.1002/pds.70042 39532529

[12] Raventós B, Prieto-Alhambra D. Real-world evidence for regulatory purposes: the example of DARWIN EU^®^. Farm Hosp. 2025;49(2):62–4. 10.1016/j.farma.2025.02.011 40102084

[13] Leinonen MK, Miettinen J, Heikkinen S, Pitkäniemi J, Malila N. Quality measures of the population-based Finnish Cancer Registry indicate sound data quality for solid malignant tumours. Eur J Cancer. 2017;77:31–9. 10.1016/j.ejca.2017.02.017 28350996

[14] Sanden GAC van D, Coebergh JWW, Schouten LJ, Visser O, Leeuwen FE van. Cancer incidence in the Netherlands in 1989 and 1990: first results of the nationwide Netherlands cancer registry. Eur J Cancer. 1995;31(11):1822–9. 10.1016/0959-8049(95)00355-M 8541107

[15] Chaplin A, Archangelidi O, Hagberg K, Neasham D, Kafatos G. Comparison of epidemiological estimates for 19 cancer types using electronic health record databases in England: an analysis of CPRD aurum and CPRD GOLD Databases with linked hospital episode statistics and cancer registry data. CLEP. 2025;17:1025–38. 10.2147/CLEP.S558429 PMC12697109 41394099

[16] Blacketer C, Defalco FJ, Ryan PB, Rijnbeek PR. Increasing trust in real-world evidence through evaluation of observational data quality. J Am Med Inform Assoc. 2021;28(10):2251–7. 10.1093/jamia/ocab132 34313749 PMC8449628

[17] Reich C, Ostropolets A, Ryan P, Rijnbeek P, Schuemie M, Davydov A, et al. OHDSI Standardized vocabularies – a large-scale centralized reference ontology for international data harmonization. J Am Med Inform Assoc. 2024;31(3):583–90. 10.1093/jamia/ocad247 38175665 PMC10873827

[18] Hripcsak G, Albers DJ. High-fidelity phenotyping: richness and freedom from bias. J Am Medi Inform Assoc. 2018;25(3):289–94. 10.1093/jamia/ocx110 PMC7282504 29040596

[19] Catala M, Guo Y, Du M, Lopez-Guell K, Burn E, Mercade-Besora N, et al. PatientProfiles: identify characteristics of patients in the OMOP common data model. 2025 [cited 2025 Apr 08]. Available from: https://cran.r-project.org/web/packages/PatientProfiles/index.html

[20] López-Güell K, Burn E, Catala M, Li X, Newby D, Mercade-Besora N. CohortSurvival: estimate survival from common data model cohorts. 2025 [cited 2025 Apr 08]. Available from: https://cran.r-project.org/web/packages/CohortSurvival/index.html

[21] Hurwitz HI, Dowlati A, Saini S, Savage S, Suttle AB, Gibson DM, et al. Phase I trial of pazopanib in patients with advanced cancer. Clin Cancer Res. 2009;15(12):4220–7. 10.1158/1078-0432.CCR-08-2740 19509175

[22] Schuetze SM, Wathen JK, Lucas DR, Choy E, Samuels BL, Staddon AP, et al. SARC009: phase 2 study of dasatinib in patients with previously treated, high‐grade, advanced sarcoma. Cancer. 2016;122(6):868–74. 10.1002/cncr.29858 26710211

[23] Rock A, Ali S, Chow WA. Systemic therapy for chondrosarcoma. Curr Treat Options Oncol. 2022;23(2):199–209. 10.1007/s11864-022-00951-7 35190971

[24] Duffaud F, Italiano A, Bompas E, Rios M, Penel N, Mir O, et al. Efficacy and safety of regorafenib in patients with metastatic or locally advanced chondrosarcoma: results of a non-comparative, randomised, double-blind, placebo controlled, multicentre phase II study. Eur J Cancer. 2021;150:108–18. 10.1016/j.ejca.2021.03.039 33895682

[25] Wagner MJ, Ricciotti RW, Mantilla J, Loggers ET, Pollack SM, Cranmer LD. Response to PD1 inhibition in conventional chondrosarcoma. J Immunother Cancer. 2018;6(1):94. 10.1186/s40425-018-0413-z 30253794 PMC6156853

[26] Somaiah N, Conley AP, Parra ER, Lin H, Amini B, Solis Soto L, et al. Durvalumab plus tremelimumab in advanced or metastatic soft tissue and bone sarcomas: a single-centre phase 2 trial. Lancet Oncol. 2022;23(9):1156–66. 10.1016/S1470-2045(22)00392-8 35934010

[27] Trama A, Bernasconi A, Cañete A, Carulla M, Daubisse-Marliac L, Rossi S, et al. Incidence and survival of rare adult solid cancers in Europe (EUROCARE-6): a population-based study. Eur J Cancer. 2025;214:115147. 10.1016/j.ejca.2024.115147 39647345

[28] Trama A, Lasalvia P, Stark D, McCabe MG, Van Der Graaf W, Gaspar N, et al. Incidence and survival of European adolescents and young adults diagnosed with sarcomas: EUROCARE-6 results. Eur J Cancer. 2025;217:115212. 10.1016/j.ejca.2024.115212 39848113

[29] Kondo H, Ogura K, Morizane C, Satake T, Iwata S, Toda Y, et al. Chondrosarcoma in Japan: an analytic study using population-based National Cancer Registry. Jpn J Clin Oncol. 2025:5(5):490–7. 10.1093/jjco/hyaf024 39893587

[30] Nie Z, Lu Q, Peng H. Prognostic factors for patients with chondrosarcoma: a survival analysis based on the Surveillance, Epidemiology, and End Results (SEER) database (1973–2012). J Bone Oncol. 2018;13:55–61. 10.1016/j.jbo.2018.09.003 30591858 PMC6303539

[31] Miyazaki T, Oike N, Ariizumi T, Murayama Y, Hatano H, Yamagishi T, et al. A retrospective study of post-radiation sarcoma from three institutions in Japan with a review of the literature. Nagoya J Med Sci. 2025;87(4):747–65. 10.18999/nagjms.87.4.747 41550183 PMC12805126

[32] Peretz Soroka H, Vora T, Noujaim J, Marcoux N, Cohen‐Gogo S, Alcindor T, et al. Real‐world experience of tyrosine kinase inhibitors in children, adolescents and adults with relapsed or refractory bone tumours: a Canadian Sarcoma Research and Clinical Collaboration (CanSaRCC) study. Cancer Med. 2023;12(18):18872–81. 10.1002/cam4.6515 37724607 PMC10557866

[33] Tap WD, Cote GM, Burris H, Gore L, Elias A, Beeram M, et al. Phase I study of the mutant IDH1 inhibitor ivosidenib: long-term safety and clinical activity in patients with conventional chondrosarcoma. Clin Cancer Res. 2025;31(11):2108–14. 10.1158/1078-0432.CCR-24-4128 40100120 PMC12130799

