# Supplementary material for: Characteristics, treatment and survival of patients with chondrosarcoma in five European countries: a DARWIN EU® cohort study
Source: Acta Oncol. 2026 Mar 10;65:45117. doi: 10.2340/1651-226X.2026.45117 (PMC12988406; doi:10.2340/1651-226X.2026.45117)
Supplement: Supplementary file 1 [file AO-65-45117-s1.pdf]

Supplementary material has been published as submitted. It has not been copyedited, or typeset by Acta Oncologica

Supplementary material

Characteristics, treatment and survival of patients with chondrosarcoma in five European countries: DARWIN EU cohort study

Anton Barchuk, Cesar Barboza, Julieta Politi, Berta Raventós, Peter Prinsen, Jelle Evers, Vincent KY Ho, Michiel AJ van de Sande, Eric Fey, Kimmo Porkka, Anna Hammais, Tiina Wahlfors, Tuomo Nieminen, Toni Lehtonen, Antonella Delmestri, Guillaume Verdy, Romain Griffier, Airam de Burgos-González, Ana Llorente-Garcia, Cristina Justo-Astorgano, Miguel-Angel Macia-Martinez, Anja Schiel, Olli Tenhunen, Ross Brennan, Alexandra Pacurariu, Ross Williams, Katia Verhamme and Talita Duarte-Salles

Description of Data Sources

Base de Datos para la Investigación Farmacoepidemiológica en el Ámbito Público (BIFAP), Spain

BIFAP is a longitudinal population-based data source of medical patient records of the Spanish National Health Service from several participating Regions throughout Spain. The population currently included represents 36% of the total Spanish population. The Spanish National Health Service provides universal access to health services through the Regional Healthcare Services. Primary care physicians, both General practitioners (GPs) and paediatricians, have a central role. They act as gatekeepers of the system and exchange information with other levels of care to ensure continuity. Most (98.9%) of the population is registered with a primary care physician and, in addition, most drug prescriptions are written at the primary care level. BIFAP includes a collection of databases linked at individual patient levels. The main one is the Primary care Database, given the central role of primary care physicians in the Spanish National Health Service. There are additional important structural databases like the medicines dispensed at community pharmacies and the patients' hospital diagnosis at discharge linked to BIFAP. Linkage to SARS-CoV-2 diagnostics test and COVID-19 vaccination registries are also included. Additional databases are also linked for a subset of patients (hospital pharmacy, cause of death registry). BIFAP program is a non-profit program financed by the Spanish Agency of Medicines and Medical Devices, a government agency belonging to the Ministry of Health in collaboration with the regional health authorities. The main use of BIFAP is for research purposes to evaluate the adverse and beneficial effects of drugs and drug utilisation patterns in the general population under real conditions of use.

Clinical Data Warehouse of Bordeaux University Hospital (CDWBordeaux), France

The clinical data warehouse of the Bordeaux University Hospital comprises Electronic Health Records (EHR) on more than 2 million patients, with data collection starting in 2005 (27). The hospital complex is made up of three main sites and comprises a total of 3,041 beds (2021 figures). The database currently holds information about patient characteristics (demographics), visits (inpatient and outpatient), conditions and procedures (billing codes), drugs (outpatient prescriptions and inpatient orders and administrations), measurements (laboratory tests and vital signs) and dates of death (in or out-hospital death). The hospital production information system data are loaded daily into a CDW in i2b2 format. A specific Extract, Transform & Load process from i2b2 to OMOP has been set up to standardise the data in OMOP-CDM format. Currently, this mapping process is launched manually when needed. The data is integrated into the OMOP CDM version and is stored in Oracle version 19c.

Clinical Practice Research Datalink GOLD (CPRD GOLD), UK

The Clinical Practice Research Datalink (CPRD) GOLD is a database of anonymised EHRs from GP clinics in the UK that use the Vision® software system for their management (28).

98% of the population in the United Kingdom (UK) is registered with a GP primarily responsible for non-emergency care and referrals to secondary care as needed. Participating GPs provide CPRD EHR for all registered patients who did not specifically request to opt out of data sharing. GOLD currently contains data from 985 up-to-standard GP practices and for nearly 21 million patients whose data quality is routinely assessed by CPRD as acceptable for clinical research. More than 3 million of these patients are alive and registered in 401 contributing practices. Based on the latest UK population estimates from the UK Office of National Statistics, GOLD covers 4.6% of the current UK population and includes 4.9% of currently contributing GP practices. GOLD contains data from all four UK constituent countries, and the current regional distribution of its GP practices is 5.7% in England, 55.6% in Scotland, 28.4% in Wales, and 10.2% in Northern Ireland (May 2022). GOLD data include the patient's demographic, biological measurements, clinical symptoms and diagnoses, referrals to specialists/hospital and their outcomes, laboratory tests/results, and prescribed medications. GOLD has been assessed and found broadly representative of the UK general population regarding age, gender, and ethnicity. GOLD has been widely used internationally for observational research to produce nearly 3,000 peer-reviewed publications, making GOLD the most influential UK clinical database so far.

Finnish Care Register for Health Care (FinOMOP - HILMO), Finland
The Finnish Care Register for Health Care (fi: Hoitoilmoitusrekisteri) continues the former Hospital Discharge Register, which originally gathered data on patients discharged from hospitals (29). The Care Register has comprehensive data on the use of services and service users from Finnish public inpatient and outpatient primary and specialised care nationwide. Since 1998, the register has covered public outpatient and inpatient specialised care and private inpatient care (TerveysHilmo). Since 2011, the register has covered public primary care (AvoHilmo). Since 2020, the register has covered private outpatient care and occupational care. The CDM is currently produced from the data collection on inpatient and outpatient specialised care (TerveysHilmo) and is limited to observation periods commencing after 01/01/2015. The inclusion of data collected before 2015 is also being planned. The National Population Registry is also used as a source for the CDM database. The National Population Registry data forms the basis for forming the patient population. This ensures up-to-date location (municipality of residence) of patients and complete death occurrences (although not the cause of death). Using the complete population as a basis for the person table also facilitates calculations on a population level, e.g. incidence rates. HILMO database is used to assess the quality of cancer registry data in Finland (30).

Hospital District of Helsinki and Uusimaa (FinOMOP - HUS), Finland
The HUS data lake is a comprehensive, integrated data source derived in real-time from all patients who visit the HUS hospitals and receive treatment (31). HUS is responsible for specialised healthcare in Finland's Uusimaa region and the treatment of many rare and severe diseases, which are nationally centralised to HUS. HUS's catchment area covers about 2.2 million people. In 2023, there were 2.43 million booked appointments and 255,896 emergency department visits for specialist medical care. A total of 691,702 patients received any treatment in HUS specialist medical care and at emergency departments, and 86,849 surgical procedures were performed. All visits, examinations, laboratory tests, procedures, and treatments are recorded in the HUS IT systems and integrated into the data lake. The data lake stores decades of clinical information in digital format, and data from both past and current source systems is available.

Netherlands Cancer Registry (NCR), the Netherlands
The NCR compiles clinical data of all individuals newly diagnosed with cancer in the Netherlands (32). Cancer registration clerks have been registering newly diagnosed cancer patients on a national basis since 1989, with a total of 3 million patients included. Data since 1992 is available in the OMOP-CDM. Over the past 35 years, this registry has provided clinicians and researchers with a wealth of clinical data (e.g., patient and tumour

characteristics, primary treatment, survival) on cancer patients of all ages. Specifically, it also comprises information on tumour staging (according to the AJCC/UICC TNM classification), tumour site (topography) and morphology (histology) (according to the WHO International Classification of Diseases for Oncology (ICD-O-3)), and treatment received directly after diagnosis (within the first 9 months after diagnosis). Overall, patients are followed up for less than one year, except for death, collected any time after diagnosis. See https://iknl.nl/en for more information.

References

1. Ferté T, Jouhet V, Griffier R, Hejblum BP, Thiébaut R, Bordeaux University Hospital Covid-19 Crisis Task Force. The benefit of augmenting open data with clinical data-warehouse EHR for forecasting SARS-CoV-2 hospitalizations in Bordeaux area, France. *JAMIA Open*. 2022;5(4):ooac086. doi:10.1093/jamiaopen/ooac086

2. Sanchez-Santos MT, Axson E, Dedman D, Delmestri A, Data Resource Profile Update: CPRD GOLD. *Int J Epidemiol.* 2025. In press, https://doi.org/10.1093/ije/dyaf077

3. Sund R. Quality of the Finnish Hospital Discharge Register: A systematic review. *Scand J Public Health*. 2012;40(6):505-515. doi:10.1177/1403494812456637

4. Leinonen MK, Anttila A, Malila N, Dillner J, Forslund O, Nieminen P. Type- and age-specific distribution of human papillomavirus in women attending cervical cancer screening in Finland. *Br J Cancer*. 2013;109(11):2941-2950. doi:10.1038/bjc.2013.647

5. Vikkula J, Uusi-Rauva K, Ranki T, Toppila I, Aalto-Setälä M, Pousar K, Vassilev L, Porkka K, Silvennoinen R, Brück O. Real-world Evidence of Multiple Myeloma Treated from 2013 to 2019 in the Hospital District of Helsinki and Uusimaa, Finland. *Future Oncol*. 2023;19(30):2029-2043. doi:10.2217/fon-2023-0120

6. Sanden GAC van D, Coebergh JWW, Schouten LJ, Visser O, Leeuwen FE van. Cancer incidence in the Netherlands in 1989 and 1990: First results of the nationwide Netherlands cancer registry. *Eur J Cancer*. 1995;31(11):1822-1829. doi:10.1016/0959-8049(95)00355-M

Table S1: Description of data sources

Country	Name of Database	Justification for Inclusion	Health Care setting	Type of Data	Number of active subjects	Data lock for the last update
ES	BIFAP	Information on chondrosarcoma patient characteristics at the time of diagnosis and hospital registry data with high-quality information on chondrosarcoma diagnoses, mortality.	Primary care – GPs, community pharmacists, primary care specialists (e.g. paediatricians), hospital IP care	EHR hospital, claims	22.0 M	2024-03-26
FR	CDWBordeaux	Hospital registry data with high-quality information on chondrosarcoma diagnoses, mortality, and treatment.	Secondary care – specialists (ambulatory or hospital OP care), hospital IP care,	EHR hospital, claims, Biobank	2.2 M	2024-01-17

Country	Name of Database	Justification for Inclusion	Health Care setting	Type of Data	Number of active subjects	Data lock for the last update
GB	CPRD GOLD	Information on chondrosarcoma patient characteristics at the time of diagnosis and information on mortality.	Primary care – GPs, primary care specialists (e.g. paediatricians)	EHR	17.3 M	2024-03-22
FI	FinOMOP - HILMO	Nation-wide hospital registry data with high-quality information on chondrosarcoma diagnoses and mortality	Secondary care – specialists (ambulatory or hospital OP care), hospital IP care	EHR, Registries	7.3 M	2024-02-12
FI	FinOMOP - HUS	Hospital registry data with high-quality information on chondrosarcoma diagnoses, mortality, and treatment.	Secondary care – specialists (ambulatory or hospital OP care), hospital IP care	EHR	3.5 M	2024-02-16
NL	NCR	Nationwide cancer registry data with high-quality information on chondrosarcoma diagnoses, mortality, and treatment.	Cancer registry, primary care, secondary care (ambulatory and hospital care)	Registries	2.5 M	2024-01-01 (follow-up) 2022-12-31 (registration)

IP = inpatient, OP = outpatient, EHR = electronic health records, OT = other, NA = not applicable, GP = general practitioner
ES = Spain, FR = France, GB = the United Kingdom of Great Britain and Northern Ireland, FI = Finland, NL = the Netherlands.

Table S2 Classification, codes and and concept IDs used to identify chondrosarcoma patients.

Id	Code	Name	Class	Vocabulary	Comments
40481938	443520009	Chondrosarcoma	Clinical Finding	SNOMED	
37151900	1163016002	Chondrosarcoma	Morph Abnormality	SNOMED	
40486574	447792005	Chondrosarcoma of bone	Clinical Finding	SNOMED	
607436	1153424004	Chondrosarcoma of bone of pelvic wall	Clinical Finding	SNOMED	
607437	1153425003	Chondrosarcoma of clavicle	Clinical Finding	SNOMED	
607434	1153422000	Chondrosarcoma of mandible	Clinical Finding	SNOMED	
607435	1153423005	Chondrosarcoma of rib	Clinical Finding	SNOMED	
607438	1153426002	Chondrosarcoma of skull	Clinical Finding	SNOMED	
607433	1153421007	Chondrosarcoma of sternum	Clinical Finding	SNOMED	
607439	1153427006	Chondrosarcoma of vertebral column	Clinical Finding	SNOMED	
45773107	703699000	Chondrosarcoma, grade 2	Morph Abnormality	SNOMED	
45766523	703700004	Chondrosarcoma, grade 3	Morph Abnormality	SNOMED	

36534641	9220/3-C31.9	Chondrosarcoma, NOS, of accessory sinus, NOS	ICDO Condition	ICDO3	
36523756	9220/3-C72.4	Chondrosarcoma, NOS, of acoustic nerve	ICDO Condition	ICDO3	
42511630	9220/3-C11.3	Chondrosarcoma, NOS, of anterior wall of nasopharynx	ICDO Condition	ICDO3	
42512935	9220/3-C60.2	Chondrosarcoma, NOS, of body of penis	ICDO Condition	ICDO3	
36524031	9220/3-C41.0	Chondrosarcoma, NOS, of bones of skull and face and associated joints	ICDO Condition	ICDO3	
36551284	9220/3-C71.7	Chondrosarcoma, NOS, of brain stem	ICDO Condition	ICDO3	
44499912	9220/3-C71.9	Chondrosarcoma, NOS, of brain, NOS	ICDO Condition	ICDO3	
36533603	9220/3-C72.1	Chondrosarcoma, NOS, of cauda equina	ICDO Condition	ICDO3	
36550660	9220/3-C71.6	Chondrosarcoma, NOS, of cerebellum, NOS	ICDO Condition	ICDO3	
36535191	9220/3-C70.0	Chondrosarcoma, NOS, of cerebral meninges	ICDO Condition	ICDO3	
36551471	9220/3-C71.0	Chondrosarcoma, NOS, of cerebrum	ICDO Condition	ICDO3	
42512383	9220/3-C49.0	Chondrosarcoma, NOS, of connective, Subcutaneous and other soft tissues of head, face, and neck	ICDO Condition	ICDO3	
36529266	9220/3-C49.2	Chondrosarcoma, NOS, of connective, Subcutaneous and other soft tissues of lower limb and hip	ICDO Condition	ICDO3	
36537890	9220/3-C49.5	Chondrosarcoma, NOS, of connective, Subcutaneous and other soft tissues of pelvis	ICDO Condition	ICDO3	
42511882	9220/3-C49.3	Chondrosarcoma, NOS, of connective, Subcutaneous and other soft tissues of thorax	ICDO Condition	ICDO3	
36518305	9220/3-C49.6	Chondrosarcoma, NOS, of connective, Subcutaneous and other soft tissues of trunk, NOS	ICDO Condition	ICDO3	
36538559	9220/3-C49.1	Chondrosarcoma, NOS, of connective, Subcutaneous and other soft tissues of upper limb and shoulder	ICDO Condition	ICDO3	
36527602	9220/3-C72.5	Chondrosarcoma, NOS, of cranial nerve, NOS	ICDO Condition	ICDO3	
44501747	9220/3-C67.1	Chondrosarcoma, NOS, of dome of bladder	ICDO Condition	ICDO3	
36545965	9220/3-C31.1	Chondrosarcoma, NOS, of ethmoid sinus	ICDO Condition	ICDO3	
44499586	9220/3-C71.1	Chondrosarcoma, NOS, of frontal lobe	ICDO Condition	ICDO3	

36540261	9220/3-C31.2	Chondrosarcoma, NOS, of frontal sinus	ICDO Condition	ICDO3	
36526411	9220/3-C32.0	Chondrosarcoma, NOS, of glottis	ICDO Condition	ICDO3	
36520352	9220/3-C13.1	Chondrosarcoma, NOS, of hypopharyngeal aspect of aryepiglottic fold	ICDO Condition	ICDO3	
36547692	9220/3-C13.9	Chondrosarcoma, NOS, of hypopharynx, NOS	ICDO Condition	ICDO3	
36563462	9220/3-C32.3	Chondrosarcoma, NOS, of laryngeal cartilage	ICDO Condition	ICDO3	
44502056	9220/3-C32.9	Chondrosarcoma, NOS, of larynx, NOS	ICDO Condition	ICDO3	
36548029	9220/3-C40.2	Chondrosarcoma, NOS, of long bones of lower limb and associated joints	ICDO Condition	ICDO3	
36518039	9220/3-C40.0	Chondrosarcoma, NOS, of long bones of upper limb, scapula and associated joints	ICDO Condition	ICDO3	
44499598	9220/3-C41.1	Chondrosarcoma, NOS, of mandible	ICDO Condition	ICDO3	
36549873	9220/3-C31.0	Chondrosarcoma, NOS, of maxillary sinus	ICDO Condition	ICDO3	
36527279	9220/3-C70.9	Chondrosarcoma, NOS, of meninges, NOS	ICDO Condition	ICDO3	
36544650	9220/3-C30.0	Chondrosarcoma, NOS, of nasal cavity	ICDO Condition	ICDO3	
42512891	9220/3-C11.9	Chondrosarcoma, NOS, of nasopharynx, NOS	ICDO Condition	ICDO3	
36541137	9220/3-C72.9	Chondrosarcoma, NOS, of nervous system, NOS	ICDO Condition	ICDO3	
36529737	9220/3-C71.4	Chondrosarcoma, NOS, of occipital lobe	ICDO Condition	ICDO3	
36532862	9220/3-C72.2	Chondrosarcoma, NOS, of olfactory nerve	ICDO Condition	ICDO3	
36565610	9220/3-C72.3	Chondrosarcoma, NOS, of optic nerve	ICDO Condition	ICDO3	
42512428	9220/3-C56.9	Chondrosarcoma, NOS, of ovary	ICDO Condition	ICDO3	
36535535	9220/3-C31.8	Chondrosarcoma, NOS, of overlapping lesion of accessory sinuses	ICDO Condition	ICDO3	
36532068	9220/3-C67.8	Chondrosarcoma, NOS, of overlapping lesion of bladder	ICDO Condition	ICDO3	
36529826	9220/3-C41.8	Chondrosarcoma, NOS, of overlapping lesion of bones, joints and articular cartilage	ICDO Condition	ICDO3	
36534535	9220/3-C40.8	Chondrosarcoma, NOS, of overlapping lesion of bones, joints and articular cartilage of limbs	ICDO Condition	ICDO3	

36546085	9220/3-C71.8	Chondrosarcoma, NOS, of overlapping lesion of brain	ICDO Condition	ICDO3	
36533147	9220/3-C72.8	Chondrosarcoma, NOS, of overlapping lesion of brain and central nervous system	ICDO Condition	ICDO3	
36531275	9220/3-C13.8	Chondrosarcoma, NOS, of overlapping lesion of hypopharynx	ICDO Condition	ICDO3	
36522901	9220/3-C32.8	Chondrosarcoma, NOS, of overlapping lesion of larynx	ICDO Condition	ICDO3	
36554271	9220/3-C71.3	Chondrosarcoma, NOS, of parietal lobe	ICDO Condition	ICDO3	
36529504	9220/3-C41.4	Chondrosarcoma, NOS, of pelvic bones, sacrum, coccyx and associated joints	ICDO Condition	ICDO3	
36546977	9220/3-C13.0	Chondrosarcoma, NOS, of postcricoid region	ICDO Condition	ICDO3	
36564901	9220/3-C13.2	Chondrosarcoma, NOS, of posterior wall of hypopharynx	ICDO Condition	ICDO3	
36559448	9220/3-C61.9	Chondrosarcoma, NOS, of prostate gland	ICDO Condition	ICDO3	
36538017	9220/3-C12.9	Chondrosarcoma, NOS, of pyriform sinus	ICDO Condition	ICDO3	
44503177	9220/3-C48.0	Chondrosarcoma, NOS, of retroperitoneum	ICDO Condition	ICDO3	
36526470	9220/3-C41.3	Chondrosarcoma, NOS, of rib, sternum, clavicle and associated joints	ICDO Condition	ICDO3	
36557249	9220/3-C40.3	Chondrosarcoma, NOS, of short bones of lower limb and associated joints	ICDO Condition	ICDO3	
36538913	9220/3-C40.1	Chondrosarcoma, NOS, of short bones of upper limb and associated joints	ICDO Condition	ICDO3	
36535268	9220/3-C31.3	Chondrosarcoma, NOS, of sphenoid sinus	ICDO Condition	ICDO3	
36551347	9220/3-C72.0	Chondrosarcoma, NOS, of spinal cord	ICDO Condition	ICDO3	
36556860	9220/3-C70.1	Chondrosarcoma, NOS, of spinal meninges	ICDO Condition	ICDO3	
36535371	9220/3-C32.2	Chondrosarcoma, NOS, of subglottis	ICDO Condition	ICDO3	
36518500	9220/3-C32.1	Chondrosarcoma, NOS, of supraglottis	ICDO Condition	ICDO3	
36563136	9220/3-C71.2	Chondrosarcoma, NOS, of temporal lobe	ICDO Condition	ICDO3	
44502133	9220/3-C33.9	Chondrosarcoma, NOS, of trachea	ICDO Condition	ICDO3	
36519748	9220/3-C71.5	Chondrosarcoma, NOS, of ventricle, NOS	ICDO Condition	ICDO3	
44501863	9220/3-C41.2	Chondrosarcoma, NOS, of vertebral column	ICDO Condition	ICDO3	

4028692	128775007	Clear cell chondrosarcoma	Morph Abnormality	SNOMED	Clear cell chondrosarcoma
36534545	9242/3-C47.9	Clear cell chondrosarcoma of autonomic nervous system, NOS	ICDO Condition	ICDO3	Clear cell chondrosarcoma
36535201	9242/3-C40.9	Clear cell chondrosarcoma of bone of limb, NOS	ICDO Condition	ICDO3	Clear cell chondrosarcoma
36564258	9242/3-C41.9	Clear cell chondrosarcoma of bone, NOS	ICDO Condition	ICDO3	Clear cell chondrosarcoma
36526737	9242/3-C41.0	Clear cell chondrosarcoma of bones of skull and face and associated joints	ICDO Condition	ICDO3	Clear cell chondrosarcoma
36564132	9242/3-C49.4	Clear cell chondrosarcoma of connective, Subcutaneous and other soft tissues of abdomen	ICDO Condition	ICDO3	Clear cell chondrosarcoma
36556686	9242/3-C49.0	Clear cell chondrosarcoma of connective, Subcutaneous and other soft tissues of head, face, and neck	ICDO Condition	ICDO3	Clear cell chondrosarcoma
36526745	9242/3-C49.2	Clear cell chondrosarcoma of connective, Subcutaneous and other soft tissues of lower limb and hip	ICDO Condition	ICDO3	Clear cell chondrosarcoma
36522616	9242/3-C49.5	Clear cell chondrosarcoma of connective, Subcutaneous and other soft tissues of pelvis	ICDO Condition	ICDO3	Clear cell chondrosarcoma
36533886	9242/3-C49.3	Clear cell chondrosarcoma of connective, Subcutaneous and other soft tissues of thorax	ICDO Condition	ICDO3	Clear cell chondrosarcoma
36527978	9242/3-C49.6	Clear cell chondrosarcoma of connective, Subcutaneous and other soft tissues of trunk, NOS	ICDO Condition	ICDO3	Clear cell chondrosarcoma
36526660	9242/3-C49.1	Clear cell chondrosarcoma of connective, Subcutaneous and other soft tissues of upper limb and shoulder	ICDO Condition	ICDO3	Clear cell chondrosarcoma
36539180	9242/3-C49.9	Clear cell chondrosarcoma of connective, Subcutaneous and other soft tissues, NOS	ICDO Condition	ICDO3	Clear cell chondrosarcoma
42511684	9242/3-C32.3	Clear cell chondrosarcoma of laryngeal cartilage	ICDO Condition	ICDO3	Clear cell chondrosarcoma
36535683	9242/3-C40.2	Clear cell chondrosarcoma of long bones of lower limb and associated joints	ICDO Condition	ICDO3	Clear cell chondrosarcoma
36564835	9242/3-C40.0	Clear cell chondrosarcoma of long bones of upper limb, scapula and associated joints	ICDO Condition	ICDO3	Clear cell chondrosarcoma
36537788	9242/3-C41.1	Clear cell chondrosarcoma of mandible	ICDO Condition	ICDO3	Clear cell chondrosarcoma
36537195	9242/3-C41.8	Clear cell chondrosarcoma of overlapping lesion of bones, joints and articular cartilage	ICDO Condition	ICDO3	Clear cell chondrosarcoma
36564498	9242/3-C40.8	Clear cell chondrosarcoma of overlapping lesion of bones,	ICDO Condition	ICDO3	Clear cell chondrosarcoma

		joints and articular cartilage of limbs			
36538216	9242/3-C49.8	Clear cell chondrosarcoma of overlapping lesion of connective, subcutaneous and other soft tissues	ICDO Condition	ICDO3	Clear cell chondrosarcoma
36518295	9242/3-C47.8	Clear cell chondrosarcoma of overlapping lesion of peripheral nerves and autonomic nervous system	ICDO Condition	ICDO3	Clear cell chondrosarcoma
36530851	9242/3-C41.4	Clear cell chondrosarcoma of pelvic bones, sacrum, coccyx and associated joints	ICDO Condition	ICDO3	Clear cell chondrosarcoma
36552197	9242/3-C47.4	Clear cell chondrosarcoma of peripheral nerves and autonomic nervous system of abdomen	ICDO Condition	ICDO3	Clear cell chondrosarcoma
36556938	9242/3-C47.0	Clear cell chondrosarcoma of peripheral nerves and autonomic nervous system of head, face, and neck	ICDO Condition	ICDO3	Clear cell chondrosarcoma
36559564	9242/3-C47.2	Clear cell chondrosarcoma of peripheral nerves and autonomic nervous system of lower limb and hip	ICDO Condition	ICDO3	Clear cell chondrosarcoma
36541972	9242/3-C47.5	Clear cell chondrosarcoma of peripheral nerves and autonomic nervous system of pelvis	ICDO Condition	ICDO3	Clear cell chondrosarcoma
36520663	9242/3-C47.3	Clear cell chondrosarcoma of peripheral nerves and autonomic nervous system of thorax	ICDO Condition	ICDO3	Clear cell chondrosarcoma
36550393	9242/3-C47.6	Clear cell chondrosarcoma of peripheral nerves and autonomic nervous system of trunk, NOS	ICDO Condition	ICDO3	Clear cell chondrosarcoma
36527454	9242/3-C47.1	Clear cell chondrosarcoma of peripheral nerves and autonomic nervous system of upper limb and shoulder	ICDO Condition	ICDO3	Clear cell chondrosarcoma
36525914	9242/3-C41.3	Clear cell chondrosarcoma of rib, sternum, clavicle and associated joints	ICDO Condition	ICDO3	Clear cell chondrosarcoma
36541718	9242/3-C40.3	Clear cell chondrosarcoma of short bones of lower limb and associated joints	ICDO Condition	ICDO3	Clear cell chondrosarcoma
36535736	9242/3-C40.1	Clear cell chondrosarcoma of short bones of upper limb and associated joints	ICDO Condition	ICDO3	Clear cell chondrosarcoma
36523751	9242/3-C41.2	Clear cell chondrosarcoma of vertebral column	ICDO Condition	ICDO3	Clear cell chondrosarcoma
37207682	82261000000107	Conventional central chondrosarcoma tumour and germline WGS (whole genome sequencing)	Procedure	SNOMED	

4029031	128776008	Dedifferentiated chondrosarcoma	Morph Abnormality	SNOMED	Dedifferentiated chondrosarcoma
36541913	9243/3-C47.9	Dedifferentiated chondrosarcoma of autonomic nervous system, NOS	ICDO Condition	ICDO3	Dedifferentiated chondrosarcoma
36547961	9243/3-C40.9	Dedifferentiated chondrosarcoma of bone of limb, NOS	ICDO Condition	ICDO3	Dedifferentiated chondrosarcoma
36527824	9243/3-C41.9	Dedifferentiated chondrosarcoma of bone, NOS	ICDO Condition	ICDO3	Dedifferentiated chondrosarcoma
36562329	9243/3-C41.0	Dedifferentiated chondrosarcoma of bones of skull and face and associated joints	ICDO Condition	ICDO3	Dedifferentiated chondrosarcoma
36551597	9243/3-C49.4	Dedifferentiated chondrosarcoma of connective, Subcutaneous and other soft tissues of abdomen	ICDO Condition	ICDO3	Dedifferentiated chondrosarcoma
36534710	9243/3-C49.0	Dedifferentiated chondrosarcoma of connective, Subcutaneous and other soft tissues of head, face, and neck	ICDO Condition	ICDO3	Dedifferentiated chondrosarcoma
36558682	9243/3-C49.2	Dedifferentiated chondrosarcoma of connective, Subcutaneous and other soft tissues of lower limb and hip	ICDO Condition	ICDO3	Dedifferentiated chondrosarcoma
36519746	9243/3-C49.5	Dedifferentiated chondrosarcoma of connective, Subcutaneous and other soft tissues of pelvis	ICDO Condition	ICDO3	Dedifferentiated chondrosarcoma
36547274	9243/3-C49.3	Dedifferentiated chondrosarcoma of connective, Subcutaneous and other soft tissues of thorax	ICDO Condition	ICDO3	Dedifferentiated chondrosarcoma
36556041	9243/3-C49.6	Dedifferentiated chondrosarcoma of connective, Subcutaneous and other soft tissues of trunk, NOS	ICDO Condition	ICDO3	Dedifferentiated chondrosarcoma
36545734	9243/3-C49.1	Dedifferentiated chondrosarcoma of connective, Subcutaneous and other soft tissues of upper limb and shoulder	ICDO Condition	ICDO3	Dedifferentiated chondrosarcoma
36544020	9243/3-C49.9	Dedifferentiated chondrosarcoma of connective, Subcutaneous and other soft tissues, NOS	ICDO Condition	ICDO3	Dedifferentiated chondrosarcoma
36530319	9243/3-C40.2	Dedifferentiated chondrosarcoma of long bones of lower limb and associated joints	ICDO Condition	ICDO3	Dedifferentiated chondrosarcoma
36521865	9243/3-C40.0	Dedifferentiated chondrosarcoma of long bones of upper limb, scapula and associated joints	ICDO Condition	ICDO3	Dedifferentiated chondrosarcoma

36539340	9243/3-C41.1	Dedifferentiated chondrosarcoma of mandible	ICDO Condition	ICDO3	Dedifferentiated chondrosarcoma
36549870	9243/3-C41.8	Dedifferentiated chondrosarcoma of overlapping lesion of bones, joints and articular cartilage	ICDO Condition	ICDO3	Dedifferentiated chondrosarcoma
36553941	9243/3-C40.8	Dedifferentiated chondrosarcoma of overlapping lesion of bones, joints and articular cartilage of limbs	ICDO Condition	ICDO3	Dedifferentiated chondrosarcoma
36562669	9243/3-C49.8	Dedifferentiated chondrosarcoma of overlapping lesion of connective, subcutaneous and other soft tissues	ICDO Condition	ICDO3	Dedifferentiated chondrosarcoma
36534973	9243/3-C47.8	Dedifferentiated chondrosarcoma of overlapping lesion of peripheral nerves and autonomic nervous system	ICDO Condition	ICDO3	Dedifferentiated chondrosarcoma
36549134	9243/3-C41.4	Dedifferentiated chondrosarcoma of pelvic bones, sacrum, coccyx and associated joints	ICDO Condition	ICDO3	Dedifferentiated chondrosarcoma
36545787	9243/3-C47.4	Dedifferentiated chondrosarcoma of peripheral nerves and autonomic nervous system of abdomen	ICDO Condition	ICDO3	Dedifferentiated chondrosarcoma
36563241	9243/3-C47.0	Dedifferentiated chondrosarcoma of peripheral nerves and autonomic nervous system of head, face, and neck	ICDO Condition	ICDO3	Dedifferentiated chondrosarcoma
36559047	9243/3-C47.2	Dedifferentiated chondrosarcoma of peripheral nerves and autonomic nervous system of lower limb and hip	ICDO Condition	ICDO3	Dedifferentiated chondrosarcoma
36529873	9243/3-C47.5	Dedifferentiated chondrosarcoma of peripheral nerves and autonomic nervous system of pelvis	ICDO Condition	ICDO3	Dedifferentiated chondrosarcoma
36530294	9243/3-C47.3	Dedifferentiated chondrosarcoma of peripheral nerves and autonomic nervous system of thorax	ICDO Condition	ICDO3	Dedifferentiated chondrosarcoma
36526712	9243/3-C47.6	Dedifferentiated chondrosarcoma of peripheral nerves and autonomic nervous system of trunk, NOS	ICDO Condition	ICDO3	Dedifferentiated chondrosarcoma
36534410	9243/3-C47.1	Dedifferentiated chondrosarcoma of peripheral nerves and autonomic nervous system of upper limb and shoulder	ICDO Condition	ICDO3	Dedifferentiated chondrosarcoma
36563608	9243/3-C41.3	Dedifferentiated chondrosarcoma of rib, sternum, clavicle and associated joints	ICDO Condition	ICDO3	Dedifferentiated chondrosarcoma
36541869	9243/3-C40.3	Dedifferentiated chondrosarcoma of short bones	ICDO Condition	ICDO3	Dedifferentiated chondrosarcoma

		of lower limb and associated joints			
36524419	9243/3-C40.1	Dedifferentiated chondrosarcoma of short bones of upper limb and associated joints	ICDO Condition	ICDO3	Dedifferentiated chondrosarcoma
36561917	9243/3-C41.2	Dedifferentiated chondrosarcoma of vertebral column	ICDO Condition	ICDO3	Dedifferentiated chondrosarcoma
4094509	26211003	Juxtacortical chondrosarcoma	Morph Abnormality	SNOMED	
4209580	56565002	Mesenchymal chondrosarcoma	Morph Abnormality	SNOMED	Mesenchymal chondrosarcoma
36567464	9240/3-C31.9	Mesenchymal chondrosarcoma of accessory sinus, NOS	ICDO Condition	ICDO3	Mesenchymal chondrosarcoma
36537160	9240/3-C47.9	Mesenchymal chondrosarcoma of autonomic nervous system, NOS	ICDO Condition	ICDO3	Mesenchymal chondrosarcoma
36526302	9240/3-C40.9	Mesenchymal chondrosarcoma of bone of limb, NOS	ICDO Condition	ICDO3	Mesenchymal chondrosarcoma
36558815	9240/3-C41.9	Mesenchymal chondrosarcoma of bone, NOS	ICDO Condition	ICDO3	Mesenchymal chondrosarcoma
36522746	9240/3-C41.0	Mesenchymal chondrosarcoma of bones of skull and face and associated joints	ICDO Condition	ICDO3	Mesenchymal chondrosarcoma
36529880	9240/3-C70.0	Mesenchymal chondrosarcoma of cerebral meninges	ICDO Condition	ICDO3	Mesenchymal chondrosarcoma
36532686	9240/3-C49.4	Mesenchymal chondrosarcoma of connective, Subcutaneous and other soft tissues of abdomen	ICDO Condition	ICDO3	Mesenchymal chondrosarcoma
36547128	9240/3-C49.0	Mesenchymal chondrosarcoma of connective, Subcutaneous and other soft tissues of head, face, and neck	ICDO Condition	ICDO3	Mesenchymal chondrosarcoma
36541806	9240/3-C49.2	Mesenchymal chondrosarcoma of connective, Subcutaneous and other soft tissues of lower limb and hip	ICDO Condition	ICDO3	Mesenchymal chondrosarcoma
36526673	9240/3-C49.5	Mesenchymal chondrosarcoma of connective, Subcutaneous and other soft tissues of pelvis	ICDO Condition	ICDO3	Mesenchymal chondrosarcoma
36553616	9240/3-C49.3	Mesenchymal chondrosarcoma of connective, Subcutaneous and other soft tissues of thorax	ICDO Condition	ICDO3	Mesenchymal chondrosarcoma
36538170	9240/3-C49.6	Mesenchymal chondrosarcoma of connective, Subcutaneous and other soft tissues of trunk, NOS	ICDO Condition	ICDO3	Mesenchymal chondrosarcoma
36530577	9240/3-C49.1	Mesenchymal chondrosarcoma of connective, Subcutaneous and other soft tissues of upper limb and shoulder	ICDO Condition	ICDO3	Mesenchymal chondrosarcoma

36561252	9240/3-C49.9	Mesenchymal chondrosarcoma of connective, Subcutaneous and other soft tissues, NOS	ICDO Condition	ICDO3	Mesenchymal chondrosarcoma
36523867	9240/3-C31.1	Mesenchymal chondrosarcoma of ethmoid sinus	ICDO Condition	ICDO3	Mesenchymal chondrosarcoma
36555967	9240/3-C31.2	Mesenchymal chondrosarcoma of frontal sinus	ICDO Condition	ICDO3	Mesenchymal chondrosarcoma
36554653	9240/3-C40.2	Mesenchymal chondrosarcoma of long bones of lower limb and associated joints	ICDO Condition	ICDO3	Mesenchymal chondrosarcoma
36561410	9240/3-C40.0	Mesenchymal chondrosarcoma of long bones of upper limb, scapula and associated joints	ICDO Condition	ICDO3	Mesenchymal chondrosarcoma
36520760	9240/3-C41.1	Mesenchymal chondrosarcoma of mandible	ICDO Condition	ICDO3	Mesenchymal chondrosarcoma
44501291	9240/3-C31.0	Mesenchymal chondrosarcoma of maxillary sinus	ICDO Condition	ICDO3	Mesenchymal chondrosarcoma
36558448	9240/3-C70.9	Mesenchymal chondrosarcoma of meninges, NOS	ICDO Condition	ICDO3	Mesenchymal chondrosarcoma
36535126	9240/3-C30.0	Mesenchymal chondrosarcoma of nasal cavity	ICDO Condition	ICDO3	Mesenchymal chondrosarcoma
730576	9240/3-C72.9	Mesenchymal chondrosarcoma of nervous system, NOS	ICDO Condition	ICDO3	Mesenchymal chondrosarcoma
36559413	9240/3-C31.8	Mesenchymal chondrosarcoma of overlapping lesion of accessory sinuses	ICDO Condition	ICDO3	Mesenchymal chondrosarcoma
36554862	9240/3-C41.8	Mesenchymal chondrosarcoma of overlapping lesion of bones, joints and articular cartilage	ICDO Condition	ICDO3	Mesenchymal chondrosarcoma
36521506	9240/3-C40.8	Mesenchymal chondrosarcoma of overlapping lesion of bones, joints and articular cartilage of limbs	ICDO Condition	ICDO3	Mesenchymal chondrosarcoma
36558095	9240/3-C49.8	Mesenchymal chondrosarcoma of overlapping lesion of connective, subcutaneous and other soft tissues	ICDO Condition	ICDO3	Mesenchymal chondrosarcoma
36549191	9240/3-C47.8	Mesenchymal chondrosarcoma of overlapping lesion of peripheral nerves and autonomic nervous system	ICDO Condition	ICDO3	Mesenchymal chondrosarcoma
44501105	9240/3-C71.3	Mesenchymal chondrosarcoma of parietal lobe	ICDO Condition	ICDO3	Mesenchymal chondrosarcoma
36549848	9240/3-C41.4	Mesenchymal chondrosarcoma of pelvic bones, sacrum, coccyx and associated joints	ICDO Condition	ICDO3	Mesenchymal chondrosarcoma
36558215	9240/3-C47.4	Mesenchymal chondrosarcoma of peripheral nerves and autonomic nervous system of abdomen	ICDO Condition	ICDO3	Mesenchymal chondrosarcoma
36556153	9240/3-C47.0	Mesenchymal chondrosarcoma of peripheral nerves and autonomic nervous system of head, face, and neck	ICDO Condition	ICDO3	Mesenchymal chondrosarcoma

36535054	9240/3-C47.2	Mesenchymal chondrosarcoma of peripheral nerves and autonomic nervous system of lower limb and hip	ICDO Condition	ICDO3	Mesenchymal chondrosarcoma
36553329	9240/3-C47.5	Mesenchymal chondrosarcoma of peripheral nerves and autonomic nervous system of pelvis	ICDO Condition	ICDO3	Mesenchymal chondrosarcoma
36552040	9240/3-C47.3	Mesenchymal chondrosarcoma of peripheral nerves and autonomic nervous system of thorax	ICDO Condition	ICDO3	Mesenchymal chondrosarcoma
36556462	9240/3-C47.6	Mesenchymal chondrosarcoma of peripheral nerves and autonomic nervous system of trunk, NOS	ICDO Condition	ICDO3	Mesenchymal chondrosarcoma
36523227	9240/3-C47.1	Mesenchymal chondrosarcoma of peripheral nerves and autonomic nervous system of upper limb and shoulder	ICDO Condition	ICDO3	Mesenchymal chondrosarcoma
42512942	9240/3-C48.0	Mesenchymal chondrosarcoma of retroperitoneum	ICDO Condition	ICDO3	Mesenchymal chondrosarcoma
36565209	9240/3-C41.3	Mesenchymal chondrosarcoma of rib, sternum, clavicle and associated joints	ICDO Condition	ICDO3	Mesenchymal chondrosarcoma
36526023	9240/3-C40.3	Mesenchymal chondrosarcoma of short bones of lower limb and associated joints	ICDO Condition	ICDO3	Mesenchymal chondrosarcoma
36562445	9240/3-C40.1	Mesenchymal chondrosarcoma of short bones of upper limb and associated joints	ICDO Condition	ICDO3	Mesenchymal chondrosarcoma
36531428	9240/3-C31.3	Mesenchymal chondrosarcoma of sphenoid sinus	ICDO Condition	ICDO3	Mesenchymal chondrosarcoma
36548053	9240/3-C70.1	Mesenchymal chondrosarcoma of spinal meninges	ICDO Condition	ICDO3	Mesenchymal chondrosarcoma
36557713	9240/3-C41.2	Mesenchymal chondrosarcoma of vertebral column	ICDO Condition	ICDO3	Mesenchymal chondrosarcoma
37207540	62511000000105	Mesenchymal chondrosarcoma tumour and germline WGS (whole genome sequencing)	Procedure	SNOMED	Mesenchymal chondrosarcoma
4328092	75622000	Myxoid chondrosarcoma	Morph Abnormality	SNOMED	Myxoid chondrosarcoma
36557238	9231/3-C40.9	Myxoid chondrosarcoma of bone of limb, NOS	ICDO Condition	ICDO3	Myxoid chondrosarcoma
36544751	9231/3-C41.9	Myxoid chondrosarcoma of bone, NOS	ICDO Condition	ICDO3	Myxoid chondrosarcoma
36549378	9231/3-C41.0	Myxoid chondrosarcoma of bones of skull and face and associated joints	ICDO Condition	ICDO3	Myxoid chondrosarcoma
44502584	9231/3-C71.9	Myxoid chondrosarcoma of brain, NOS	ICDO Condition	ICDO3	Myxoid chondrosarcoma
36531230	9231/3-C49.4	Myxoid chondrosarcoma of connective, Subcutaneous and other soft tissues of abdomen	ICDO Condition	ICDO3	Myxoid chondrosarcoma

36567516	9231/3-C49.0	Myxoid chondrosarcoma of connective, Subcutaneous and other soft tissues of head, face, and neck	ICDO Condition	ICDO3	Myxoid chondrosarcoma
36529383	9231/3-C49.2	Myxoid chondrosarcoma of connective, Subcutaneous and other soft tissues of lower limb and hip	ICDO Condition	ICDO3	Myxoid chondrosarcoma
36563461	9231/3-C49.5	Myxoid chondrosarcoma of connective, Subcutaneous and other soft tissues of pelvis	ICDO Condition	ICDO3	Myxoid chondrosarcoma
36529095	9231/3-C49.3	Myxoid chondrosarcoma of connective, Subcutaneous and other soft tissues of thorax	ICDO Condition	ICDO3	Myxoid chondrosarcoma
36561941	9231/3-C49.6	Myxoid chondrosarcoma of connective, Subcutaneous and other soft tissues of trunk, NOS	ICDO Condition	ICDO3	Myxoid chondrosarcoma
36533470	9231/3-C49.1	Myxoid chondrosarcoma of connective, Subcutaneous and other soft tissues of upper limb and shoulder	ICDO Condition	ICDO3	Myxoid chondrosarcoma
36535870	9231/3-C49.9	Myxoid chondrosarcoma of connective, Subcutaneous and other soft tissues, NOS	ICDO Condition	ICDO3	Myxoid chondrosarcoma
42512852	9231/3-C32.3	Myxoid chondrosarcoma of laryngeal cartilage	ICDO Condition	ICDO3	Myxoid chondrosarcoma
36540963	9231/3-C40.2	Myxoid chondrosarcoma of long bones of lower limb and associated joints	ICDO Condition	ICDO3	Myxoid chondrosarcoma
36542064	9231/3-C40.0	Myxoid chondrosarcoma of long bones of upper limb, scapula and associated joints	ICDO Condition	ICDO3	Myxoid chondrosarcoma
42512923	9231/3-C34.9	Myxoid chondrosarcoma of lung, NOS	ICDO Condition	ICDO3	Myxoid chondrosarcoma
36556500	9231/3-C41.1	Myxoid chondrosarcoma of mandible	ICDO Condition	ICDO3	Myxoid chondrosarcoma
36547671	9231/3-C41.8	Myxoid chondrosarcoma of overlapping lesion of bones, joints and articular cartilage	ICDO Condition	ICDO3	Myxoid chondrosarcoma
36557726	9231/3-C40.8	Myxoid chondrosarcoma of overlapping lesion of bones, joints and articular cartilage of limbs	ICDO Condition	ICDO3	Myxoid chondrosarcoma
36531067	9231/3-C71.8	Myxoid chondrosarcoma of overlapping lesion of brain	ICDO Condition	ICDO3	Myxoid chondrosarcoma
36540285	9231/3-C49.8	Myxoid chondrosarcoma of overlapping lesion of connective, subcutaneous and other soft tissues	ICDO Condition	ICDO3	Myxoid chondrosarcoma
36561481	9231/3-C41.4	Myxoid chondrosarcoma of pelvic bones, sacrum, coccyx and associated joints	ICDO Condition	ICDO3	Myxoid chondrosarcoma
42511997	9231/3-C38.2	Myxoid chondrosarcoma of posterior mediastinum	ICDO Condition	ICDO3	Myxoid chondrosarcoma

44500820	9231/3-C48.0	Myxoid chondrosarcoma of retroperitoneum	ICDO Condition	ICDO3	Myxoid chondrosarcoma
36533058	9231/3-C41.3	Myxoid chondrosarcoma of rib, sternum, clavicle and associated joints	ICDO Condition	ICDO3	Myxoid chondrosarcoma
36534364	9231/3-C40.3	Myxoid chondrosarcoma of short bones of lower limb and associated joints	ICDO Condition	ICDO3	Myxoid chondrosarcoma
36548525	9231/3-C40.1	Myxoid chondrosarcoma of short bones of upper limb and associated joints	ICDO Condition	ICDO3	Myxoid chondrosarcoma
44502736	9231/3-C71.5	Myxoid chondrosarcoma of ventricle, NOS	ICDO Condition	ICDO3	Myxoid chondrosarcoma
44503049	9231/3-C41.2	Myxoid chondrosarcoma of vertebral column	ICDO Condition	ICDO3	Myxoid chondrosarcoma
42513751	9242/3-NULL	Neoplasm defined only by histology: Clear cell chondrosarcoma	ICDO Condition	ICDO3	Clear cell chondrosarcoma
42513752	9243/3-NULL	Neoplasm defined only by histology: Dedifferentiated chondrosarcoma	ICDO Condition	ICDO3	Dedifferentiated chondrosarcoma
42513749	9240/3-NULL	Neoplasm defined only by histology: Mesenchymal chondrosarcoma	ICDO Condition	ICDO3	Mesenchymal chondrosarcoma
42513748	9231/3-NULL	Neoplasm defined only by histology: Myxoid chondrosarcoma	ICDO Condition	ICDO3	Myxoid chondrosarcoma
42513744	9221/3-NULL	Neoplasm defined only by histology: Periosteal chondrosarcoma	ICDO Condition	ICDO3	Periosteal chondrosarcoma
2102793	1E	NR4A3, RBF56, or TCF12 (myxoid chondrosarcoma) (Deprecated)	CPT4 Modifier	CPT4	Myxoid chondrosarcoma
36558653	9221/3-C40.9	Periosteal chondrosarcoma of bone of limb, NOS	ICDO Condition	ICDO3	Periosteal chondrosarcoma
36541445	9221/3-C41.9	Periosteal chondrosarcoma of bone, NOS	ICDO Condition	ICDO3	Periosteal chondrosarcoma
36544561	9221/3-C41.0	Periosteal chondrosarcoma of bones of skull and face and associated joints	ICDO Condition	ICDO3	Periosteal chondrosarcoma
36520008	9221/3-C32.0	Periosteal chondrosarcoma of glottis	ICDO Condition	ICDO3	Periosteal chondrosarcoma
36538806	9221/3-C32.3	Periosteal chondrosarcoma of laryngeal cartilage	ICDO Condition	ICDO3	Periosteal chondrosarcoma
36544844	9221/3-C32.9	Periosteal chondrosarcoma of larynx, NOS	ICDO Condition	ICDO3	Periosteal chondrosarcoma
36540974	9221/3-C40.2	Periosteal chondrosarcoma of long bones of lower limb and associated joints	ICDO Condition	ICDO3	Periosteal chondrosarcoma
36533938	9221/3-C40.0	Periosteal chondrosarcoma of long bones of upper limb, scapula and associated joints	ICDO Condition	ICDO3	Periosteal chondrosarcoma

36534232	9221/3-C41.1	Periosteal chondrosarcoma of mandible	ICDO Condition	ICDO3	Periosteal chondrosarcoma
36547380	9221/3-C30.0	Periosteal chondrosarcoma of nasal cavity	ICDO Condition	ICDO3	Periosteal chondrosarcoma
36539588	9221/3-C41.8	Periosteal chondrosarcoma of overlapping lesion of bones, joints and articular cartilage	ICDO Condition	ICDO3	Periosteal chondrosarcoma
36563361	9221/3-C40.8	Periosteal chondrosarcoma of overlapping lesion of bones, joints and articular cartilage of limbs	ICDO Condition	ICDO3	Periosteal chondrosarcoma
36547009	9221/3-C32.8	Periosteal chondrosarcoma of overlapping lesion of larynx	ICDO Condition	ICDO3	Periosteal chondrosarcoma
36553907	9221/3-C41.4	Periosteal chondrosarcoma of pelvic bones, sacrum, coccyx and associated joints	ICDO Condition	ICDO3	Periosteal chondrosarcoma
36530596	9221/3-C41.3	Periosteal chondrosarcoma of rib, sternum, clavicle and associated joints	ICDO Condition	ICDO3	Periosteal chondrosarcoma
36519044	9221/3-C40.3	Periosteal chondrosarcoma of short bones of lower limb and associated joints	ICDO Condition	ICDO3	Periosteal chondrosarcoma
36562042	9221/3-C40.1	Periosteal chondrosarcoma of short bones of upper limb and associated joints	ICDO Condition	ICDO3	Periosteal chondrosarcoma
36561619	9221/3-C32.2	Periosteal chondrosarcoma of subglottis	ICDO Condition	ICDO3	Periosteal chondrosarcoma
36565377	9221/3-C32.1	Periosteal chondrosarcoma of supraglottis	ICDO Condition	ICDO3	Periosteal chondrosarcoma
36545603	9221/3-C33.9	Periosteal chondrosarcoma of trachea	ICDO Condition	ICDO3	Periosteal chondrosarcoma
36544240	9221/3-C41.2	Periosteal chondrosarcoma of vertebral column	ICDO Condition	ICDO3	Periosteal chondrosarcoma
42539037	735678002	Primary chondrosarcoma of articular cartilage	Clinical Finding	SNOMED	
37109898	723844004	Primary chondrosarcoma of articular cartilage of limb	Clinical Finding	SNOMED	
37109899	723845003	Primary chondrosarcoma of articular cartilage of pelvis	Clinical Finding	SNOMED	
37109900	723847006	Primary chondrosarcoma of articular cartilage of rib	Clinical Finding	SNOMED	
42539556	735679005	Primary chondrosarcoma of bone	Clinical Finding	SNOMED	
602670	1079561000119103	Primary chondrosarcoma of bone of left foot	Clinical Finding	SNOMED	
602671	1079571000119109	Primary chondrosarcoma of bone of left hand	Clinical Finding	SNOMED	
602672	1079581000119107	Primary chondrosarcoma of bone of left lower limb	Clinical Finding	SNOMED	
602669	1079551000119100	Primary chondrosarcoma of bone of left upper limb	Clinical Finding	SNOMED	

37109897	723843005	Primary chondrosarcoma of bone of limb	Clinical Finding	SNOMED	
37018647	91081000119109	Primary chondrosarcoma of bone of lower limb	Clinical Finding	SNOMED	
37018646	91061000119100	Primary chondrosarcoma of bone of pelvis	Clinical Finding	SNOMED	
37119229	723846002	Primary chondrosarcoma of bone of rib	Clinical Finding	SNOMED	
602153	1079611000119100	Primary chondrosarcoma of bone of right foot	Clinical Finding	SNOMED	
602154	1079621000119107	Primary chondrosarcoma of bone of right hand	Clinical Finding	SNOMED	
602155	1079631000119105	Primary chondrosarcoma of bone of right lower limb	Clinical Finding	SNOMED	
602152	1079601000119103	Primary chondrosarcoma of bone of right upper limb	Clinical Finding	SNOMED	
37018644	91031000119108	Primary chondrosarcoma of bone of upper limb	Clinical Finding	SNOMED	
602673	1079591000119105	Primary chondrosarcoma of left scapula	Clinical Finding	SNOMED	
602036	683121000119103	Primary chondrosarcoma of mandible	Clinical Finding	SNOMED	
602674	1079641000119101	Primary chondrosarcoma of right scapula	Clinical Finding	SNOMED	
609187	16845381000119100	Primary chondrosarcoma of sternum	Clinical Finding	SNOMED	
602037	683131000119100	Primary chondrosarcoma of vertebral column	Clinical Finding	SNOMED	

Table S3. List for chondrosarcoma pharmacological management options

Drug	Class	Concept ID	RxNorm code
carboplatin	Chemotherapy	1344905	40048
cisplatin	Chemotherapy	1397599	2555
cyclophosphamide	Chemotherapy	1310317	3002
dactinomycin	Chemotherapy	1311443	3100
decitabine	Chemotherapy	19024728	15657
docetaxel	Chemotherapy	1315942	72962
doxorubicin	Chemotherapy	1338512	3639
etoposide	Chemotherapy	1350504	4179
gemcitabine	Chemotherapy	1314924	12574
ifosfamide	Chemotherapy	19078187	5657
irinotecan	Chemotherapy	1367268	51499
methotrexate	Chemotherapy	1305058	6851
temozolomide	Chemotherapy	1341149	37776
topotecan	Chemotherapy	1378509	57308
vincristine	Chemotherapy	1308290	11202
ivosidenib	IDH1 inhibitor	1560123	2049873
enasidenib	IDH2 inhibitor	1940332	793797

everolimus	mTOR kinase inhibitor	19011440	141704
toripalimab	PD-1 inhibitor	747052	2669406
retifanlimab	PD-1 inhibitor	1302024	2632981
dostarlimab	PD-1 inhibitor	1536789	2539967
cemiplimab	PD-1 inhibitor	35200783	2058826
pembrolizumab	PD-1 inhibitor	45775965	1547545
nivolumab	PD-1 inhibitor	45892628	1597876
avelumab	PD-L1 inhibitor	1593273	1875534
durvalumab	PD-L1 inhibitor	1594034	1919503
atezolizumab	PD-L1 inhibitor	42629079	1792776
afatinib	TK inhibitor	43533090	1430438
cabozantinib	TK inhibitor	43012292	1363268
dasatinib	TK inhibitor	1358436	475342
lapatinib	TK inhibitor	1359548	480167
lurbinectedin	TK inhibitor	1146139	2374729
pazopanib	TK inhibitor	40167554	714438
regorafenib	TK inhibitor	42903460	1312397
sorafenib	TK inhibitor	1363387	495881

Table S4. List of surgical and radiotherapy procedures

Id	Code	Name	Class	Vocabulary	Comments
4217482	81723002	Amputation	Procedure	SNOMED	Surgical procedure
4219032	397117006	Amputation of lower limb	Procedure	SNOMED	Surgical procedure
44790448	198341000000107	Anterolateral lymph nodes neck dissection	Procedure	SNOMED	Surgical procedure
4107982	256651005	Autogenous vascularized bone graft	Procedure	SNOMED	Surgical procedure
40317890	152198000	Brachytherapy	Procedure	SNOMED	Radiotherapy
4001562	120146008	Chest wall excision	Procedure	SNOMED	Surgical procedure
4193369	79095000	Complete excision	Procedure	SNOMED	Surgical procedure
4101626	25353009	Craniotomy	Procedure	SNOMED	Surgical procedure
4146273	427357007	Cryosurgery	Procedure	SNOMED	Surgical procedure
3662206	11971000224104	Dissection of lymph node	Procedure	SNOMED	Surgical procedure
4144920	307272003	Electrocautery operation	Procedure	SNOMED	Surgical procedure
4250344	73223003	Electrocoagulation	Procedure	SNOMED	Surgical procedure
4046732	122463005	Embolization procedure	Procedure	SNOMED	Surgical procedure
4279903	65801008	Excision	Procedure	SNOMED	Surgical procedure
4120961	234262008	Excision of axillary lymph node	Procedure	SNOMED	Surgical procedure
4118281	301426005	Excision of base of skull	Procedure	SNOMED	Surgical procedure
4346411	239329001	Excision of bone	Procedure	SNOMED	Surgical procedure
4120425	234253006	Excision of group of lymph nodes	Procedure	SNOMED	Surgical procedure

42872600	450591007	Excision of lesion of anterior abdominal wall	Procedure	SNOMED	Surgical procedure
4285420	68471001	Excision of lesion of bone	Procedure	SNOMED	Surgical procedure
4131910	12703009	Excision of lesion of pelvic wall	Procedure	SNOMED	Surgical procedure
4238646	58347006	Excision of lymph node	Procedure	SNOMED	Surgical procedure
4157779	370611004	Excision of malignant neoplasm	Procedure	SNOMED	Surgical procedure
4273761	367477001	Excision of mandible	Procedure	SNOMED	Surgical procedure
44803160	621421000000106	Excision of organ NOC	Procedure	SNOMED	Surgical procedure
40481912	443497002	Excision of sentinel lymph node	Procedure	SNOMED	Surgical procedure
4141448	33195004	External beam radiation therapy procedure	Procedure	SNOMED	Radiotherapy
4024005	10611004	External beam radiation therapy protons	Procedure	SNOMED	Radiotherapy
4061550	169332007	External beam radiation therapy with internal radiotherapy	Procedure	SNOMED	Radiotherapy
40480519	441799006	Intensity modulated radiation therapy	Procedure	SNOMED	Radiotherapy
4059831	169349000	Internal radiotherapy - unsealed source	Procedure	SNOMED	Radiotherapy
4120657	303577009	Interventional debulking surgery	Procedure	SNOMED	Surgical procedure
37109332	427541000119103	Intravenous radionuclide therapy	Procedure	SNOMED	Radiotherapy
4040441	228700000	Iodine 131 meta-iodobenzylguanidine therapy	Procedure	SNOMED	Radiotherapy
44813718	640441000000105	Laser excision of lesion of organ NOC	Procedure	SNOMED	Surgical procedure
4261829	35631009	Laser surgery	Procedure	SNOMED	Surgical procedure
4120958	234248001	Lateral lymph nodes neck dissection	Procedure	SNOMED	Surgical procedure
4084585	282435000	Maxillectomy	Procedure	SNOMED	Surgical procedure
40489482	448385000	Megavoltage radiation therapy using photons	Procedure	SNOMED	Radiotherapy
36684841	457561000124102	Microwave ablation	Procedure	SNOMED	Surgical procedure
4167550	418024000	Mohs surgery	Procedure	SNOMED	Surgical procedure
4003076	119705002	Nose excision	Procedure	SNOMED	Surgical procedure
4323226	428402007	Operative procedure on lumbosacral spinal structure	Procedure	SNOMED	Surgical procedure
4134732	398328001	Orbitectomy	Procedure	SNOMED	Surgical procedure
4304452	38829003	Partial excision	Procedure	SNOMED	Surgical procedure
44803149	565241000000104	Partial excision of organ NOC	Procedure	SNOMED	Surgical procedure
4251481	73850000	Pedicle graft	Procedure	SNOMED	Surgical procedure

4338944	88088007	Radical excision with lymph node dissection	Procedure	SNOMED	Surgical procedure
4161415	399315003	Radionuclide therapy	Procedure	SNOMED	Radiotherapy
4059385	168524008	Radiotherapy - intraoperative control	Procedure	SNOMED	Radiotherapy
44790293	231711000000108	Radiotherapy delivery	Procedure	SNOMED	Radiotherapy
45763838	700357007	Radium 223 brachytherapy	Procedure	SNOMED	Radiotherapy
4045162	122465003	Reconstruction procedure	Procedure	SNOMED	Surgical procedure
4280798	65854006	Reexcision	Procedure	SNOMED	Surgical procedure
46272913	711364006	Robotic assisted surgery	Procedure	SNOMED	Surgical procedure
4236996	56757003	Scraping	Procedure	SNOMED	Surgical procedure
4301351	387713003	Surgical procedure	Procedure	SNOMED	Surgical procedure
4165515	47479005	Total body irradiation	Procedure	SNOMED	Radiotherapy
4136166	413200004	Tumor destruction	Procedure	SNOMED	Surgical procedure

Table S5. Study attrition of individuals included in the study and its contribution to the study objectives.

	BIFAP	CDW Bordeaux	CPRD GOLD	FinOMOP - HILMO **	FinOMOP - HUS	NCR
Database population	22.0M	2.2M	17.3M	7.3M	3.5M	2.5M
First occurrence of chondrosarcoma identified in the database between January 1, 2010, and December 31, 2022	528	123	74	602	219	1,573
365 days of prior observation*	459	Not applied	65	Not applied	Not applied	Not applied
No prior cancer diagnosis (any, excluding non-melanoma skin cancer and non-specific bone cancer 2 months prior) before the diagnosis of chondrosarcoma	379	92	54	382	142	1449
Conventional or NOS chondrosarcoma	379	92	54	382	142	1,234 (75.2%)
Non-conventional chondrosarcoma***	Not applied	Not applied	Not applied	Not applied	Not applied	215 (14.8%)
Patient demographics	+	+	+	+	+	+
Treatment patterns	-	+	-	+	+	+
Tumor characteristics	-	-	-	-	-	+
Overall survival	+	+	+	+	+	+
Subgroup survival analysis	-	-	-	-	-	+

NOS = not otherwise specified

* A minimum of 365 days of prior observation criteria was applied only to databases that primarily collect primary care data (BIFAP and CPRD GOLD)
**Data from FinOMOP-HILMO were available only from 2011,
*** Patients with conventional and non-conventional chondrosarcoma could only be reliably distinguished in NCR.

Table S6. Procedures conducted 0-180 days after the initial diagnosis of chondrosarcoma.

	CDWBordeaux	FinOMOP - HILMO	FinOMOP - HUS	NCR
	**92**	**382**	**142**	**1449**
Radiotherapy	-	11 (2.9%)	<5	93 (6.4%)
Surgery	14 (15.2%)	249 (65.2%)	76 (53.5%)	1,288 (88.9%)

Table S7. Anatomical localisation of the tumour in patients with conventional chondrosarcoma (n=1,234) at the time of diagnosis in NCR.

		N(%)
Extremities	Long bones of upper limbs	224 (18.2%)
	Short bones of upper limbs	85 (6.9%)
	Long bones of lower limbs	510 (41.3%)
	Short bones of lower limbs	28 (2.3%)
Axial skeleton	Skull and face	97 (7.9%)
	Rib, sternum, clavicle	139 (11.3%)
	Vertebral column	29 (2.4%)
	Pelvic bones, sacrum, coccyx	98 (7.9%)

Table S8. Median survival, restricted mean survival time and overall 1, 3, 5, and 10-year survival probability of patients with chondrosarcoma in NCR by stage, anatomical site, and grade

	Number of patients	Deaths	Median survival (95% CI)	Restricted mean survival (SE)	1 Year survival, % (95% CI)	3 year survival, % (95% CI)	5 year survival, % (95% CI)	10 year survival, % (95% CI)
Stage I	791	70	NR	9.5 (0.1)	99 (98, 100)	97 (96, 98)	95 (94, 97)	90 (88, 92)
Stage IA	680	51	NR	9.6 (0.1)	99 (99, 100)	98 (97, 99)	96 (95, 98)	91 (89, 94)
Stage IB	108	19	NR	8.8 (0.3)	96 (93, 100)	93 (89, 98)	88 (82, 95)	80 (72, 89)
Stage II	330	81	NR	7.9 (0.2)	91 (87, 94)	83 (79, 88)	77 (73, 83)	66 (59, 73)
Stage IIA	206	35	NR	8.7 (0.2)	96 (93, 99)	90 (86, 95)	86 (81, 92)	73 (65, 82)
Stage IIB	127	47	NR (7.0, NR)	6.7 (0.4)	82 (76, 89)	72 (64, 81)	63 (54, 73)	53 (43, 65)
Stage III	21	10	4.5 (2.0, NR)	4.8 (0.9)	89 (77, 100)	60 (41, 88)	36 (16, 81)	18 (4, 90)
Stage IV	53	44	0.5 (0.4, 1.3)	1.9 (0.4)	39 (28, 55)	22 (13, 38)	15 (7, 32)	-
Stage information not available	248	37	NR	8.6 (0.2)	93 (90, 97)	88 (84, 92)	84 (79, 89)	80 (74, 87)
T1	952	94	NR	9.4 (0.1)	98 (97, 99)	96 (94, 97)	94 (92, 95)	88 (85, 90)
T2	272	96	NR	7.0 (0.3)	84 (80, 88)	75 (70, 80)	68 (62, 74)	58 (52, 65)

T3	50	14	NR (4.8, NR)	7.0 (0.7)	86 (76, 96)	72 (59, 87)	63 (48, 82)	63 (48, 82)
T4	22	9	NR (1.3, NR)	5.5 (1.1)	72 (54, 94)	51 (32, 82)	51 (32, 82)	-
T category information not available	146	30	NR	8.3 (0.3)	90 (85, 95)	85 (80, 91)	80 (74, 87)	77 (70, 85)
N0	1,185	196	NR	8.8 (0.1)	95 (93, 96)	90 (89, 92)	87 (85, 89)	79 (77, 82)
N+	19	10	1.8 (0.8, NR)	4.2 (1.1)	65 (45, 92)	45 (26, 78)	45 (26, 78)	-
N category information not available	243	37	NR	8.7 (0.2)	92 (88, 95)	89 (85, 93)	85 (81, 90)	84 (80, 89)
M0	1,371	193	NR	9.0 (0.1)	96 (95, 97)	92 (91, 94)	89 (87, 91)	83 (81, 85)
M1	58	47	0.5 (0.4, 1.0)	1.8 (0.4)	35 (25, 51)	21 (12, 36)	14 (7, 30)	-
M category information not available	17	<5	NR	9.1 (0.6)	100 (100, 100)	94 (84, 100)	88 (74, 100)	88 (74, 100)
Long bones of lower limbs	510	50	NR	9.6 (0.1)	99 (99, 100)	98 (97, 99)	95 (93, 97)	88 (85, 91)
Long bones of upper limbs	224	17	NR	9.6 (0.1)	99 (98, 100)	98 (96, 100)	95 (92, 98)	91 (87, 95)
Pelvic bones, sacrum, coccyx	98	20	NR	8.0 (0.4)	91 (86, 97)	82 (74, 90)	79 (71, 88)	75 (65, 86)
Rib, sternum, clavicle	139	24	NR	8.5 (0.3)	95 (92, 99)	87 (82, 94)	84 (78, 91)	74 (65, 85)
Short bones of lower limbs	28	<5	NR	9.8 (0.3)	100 (100, 100)	100 (100, 100)	96 (89, 100)	96 (89, 100)
Short bones of upper limbs	85	8	NR	9.2 (0.3)	96 (93, 100)	93 (87, 99)	93 (87, 99)	90 (83, 97)
Skull and face	97	7	NR	9.3 (0.2)	99 (97, 100)	97 (93, 100)	92 (86, 98)	90 (83, 98)
Vertebral column	29	9	NR (6.2, NR)	7.6 (0.7)	97 (90, 100)	89 (78, 100)	76 (60, 95)	57 (38, 85)
Grade 1 (well differentiated)	783	62	NR	9.6 (0.1)	99 (99, 100)	98 (97, 99)	96 (95, 97)	91 (89, 93)
Grade 2 (moderately differentiated)	303	53	NR	8.5 (0.2)	96 (94, 98)	91 (88, 95)	84 (80, 89)	73 (67, 80)
Grade 3 (poorly differentiated)	125	51	7.2 (4.4, NR)	6.0 (0.4)	75 (68, 83)	62 (54, 72)	55 (46, 66)	48 (37, 61)
Grade information not available	228	74	NR	6.9 (0.3)	82 (77, 88)	70 (64, 77)	68 (62, 75)	61 (53, 69)
NR - Median survival was not reached in all databases (more than 50% of patients were still alive by the end of the follow-up).								

Table S9. Median survival, restricted mean survival time and overall 1, 3, 5, 10-year survival probability of patients with non-conventional chondrosarcoma in NCR by histological type.

	N	Events	Median survival (95% CI)	Restricted mean survival (SE)	1 Year survival, % (95% CI)	3 year survival, % (95% CI)	5 year survival, % (95% CI)	10 year survival, % (95% CI)
Clear cell	11	0	NR	10.0 (0.0)	100 (100, 100)	100 (100, 100)	100 (100, 100)	100 (100, 100)
Dedifferentiated	94	64	0.9 (0.6, 1.8)	3.3 (0.4)	48 (39, 59)	32 (23, 43)	29 (20, 40)	21 (13, 37)
Mesenchymal	11	8	4.5 (1.8, NR)	4.6 (1.1)	91 (75, 100)	64 (41, 99)	33 (13, 80)	22 (7, 73)
Myxoid	71	22	NR (7.1, NR)	7.3 (0.5)	87 (80, 95)	81 (72, 91)	76 (66, 88)	58 (45, 75)
Periosteal	28	<5	NR	9.7 (0.3)	100 (100, 100)	100 (100, 100)	100 (100, 100)	94 (84, 100)
NR - Median survival was not reached in all databases (more than 50% of patients were still alive by the end of the follow-up).								

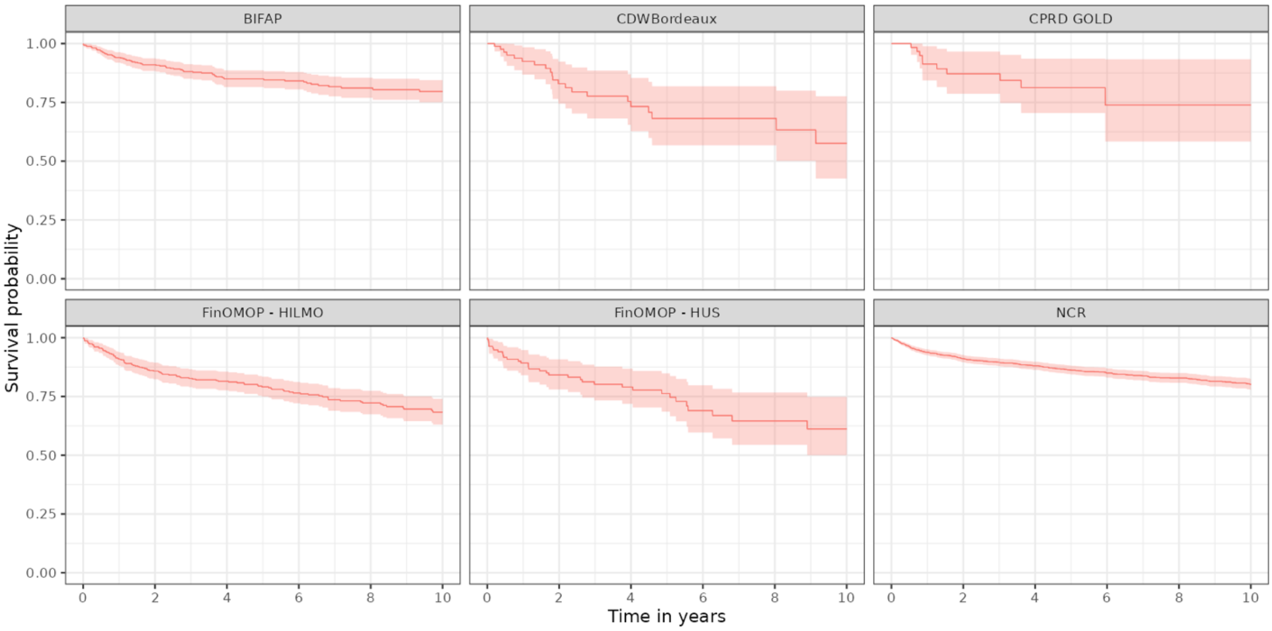

Figure S1. Overall survival of patients with chondrosarcoma by data source
